# Potential mechanism of traditional Chinese medicine intervention in gastric cancer: targeted regulation of autophagy

**DOI:** 10.3389/fphar.2025.1548672

**Published:** 2025-02-18

**Authors:** Siyuan Sun, Wenqian Yu, Guangheng Zhang, Xiangyu Li, Linjing Song, Yehan Lv, Yi Chen

**Affiliations:** ^1^ Dongzhimen Hospital, Beijing University of Chinese Medicine, Beijing, China; ^2^ First Clinical Medical College, Beijing University of Chinese Medicine, Beijing, China; ^3^ First Clinical Medical College, Shandong University of Traditional Chinese Medicine, Jinan, China; ^4^ Wangjing Hospital, China Academy of Chinese Medical Sciences, Beijing, China; ^5^ Department of Geriatrics, Third Affiliated Hospital, Beijing University of Chinese Medicine, Beijing, China

**Keywords:** autophagy, gastric cancer, traditional Chinese medicine, apoptosis, natural products

## Abstract

Gastric cancer (GC) is a prevalent malignant tumor that originates from the epithelial cells of the gastric mucosa, predominantly in the form of adenocarcinoma. Extensive research has confirmed the significant role of autophagy in the initiation, progression, and chemoresistance of GC. The potential of traditional Chinese medicine (TCM) to exert anti-tumor effects by modulating autophagy has been demonstrated, particularly in the context of GC prevention and treatment. Natural products (NPs) have great therapeutic potential in the prevention and treatment of GC by targeting autophagy-related genes and signaling pathways to intervene in the biological behaviors of gastric cancer cells such as proliferation, metastasis, invasion and death. This article describes the molecular mechanisms and key markers of tumor autophagy, the signaling pathways involved in GC-associated autophagy (PI3K/AKT/mTOR, AMPK, MAPK, p53), and summarizes the mechanism of autophagy and *Helicobacter pylori* infection in GC, how autophagy interacts with apoptotic and iron-death processes and the wide-ranging influences that these factors play in the process. Finally, this paper systematically summarizes the natural compounds of terpenoids, polyphenols, alkaloids, saponins, and polysaccharides that modulate autophagy-related signaling pathways and potential targets for the treatment of GC, and evaluates the toxic effects of NPs, providing a more compelling rationale and direction for GC therapy.

## 1 Introduction

As the fourth most common cancer in the world, data from 2020 showed that there were nearly 1.1 million new cases of GC worldwide, resulting in more than 760,000 deaths, with 75% of new GC cases and all deaths occurring in Asia (Lordick et al., 2022). Risk factors such as chronic *Helicobacter pylori* infection, Epstein-Barr virus, alcohol consumption, and high salt diet can increase the incidence of GC (Thrift et al., 2023). Bacterial or viral invasion, genetic susceptibility, and changes in the tumor microenvironment combine to accelerate the progression of GC (Figueiredo et al., 2015). However, surgical local resection has strict requirements for both the tumor and the patient’s own conditions (Spiegel et al., 2017). Immunotherapy also has some limitations in clinical application due to strong toxicity (Jin et al., 2022). In this context, accelerating the search for new drugs is crucial for early intervention in GC and addressing the health problems of GC patients worldwide.

The word “autophagy” is derived from Greek and originally means “self-eating”. Autophagy is a cellular degradation process that occurs in eukaryotic organisms, which is lysosome-dependent and essential in maintaining protein stability and organelle integrity and function (Mariño et al., 2014). Autophagy-related molecular mechanisms are highly context-specific and two-sided in tumorigenesis and progression, and their promotion or inhibition depends mainly on factors such as tumor cell type, tumor microenvironment, and disease stage (Debnath et al., 2023). In the early stage of solid tumors transitioning from precancerous lesions to malignant or invasive tumors, autophagy, as an important degradative and homeostatic mechanism, ultimately prevents cancer development by preventing toxic accumulation of damaged proteins and organelles, especially mitochondria, as well as by maintaining cellular integrity, redox balance, and protease homeostasis (Chen et al., 2021). However, in established tumor tissues, autophagy can damage normal cells and provide favorable conditions for tumor cell survival and metastasis (Xu et al., 2021a). Furthermore, modulation of autophagy enhances the efficacy of other treatment modalities such as radiotherapy, chemotherapy, and immunotherapy (Xia et al., 2021; Zhang et al., 2021). Thus, the intricate relationship between autophagy and tumors highlights the potential for targeting autophagy in cancer therapy.

NPs primarily originate from plants, animals, insects, and marine organisms in the natural world (Zhu et al., 2022). Modern pharmacology has proved that more and more NPs, especially active ingredients, play a great contribution to the prevention and treatment of diseases by virtue of their multiple targets of action, low toxicity and applicability (Hashem et al., 2022; Hu Q. et al., 2023). Autophagy plays a pivotal role in cancer progression. Studies have demonstrated that numerous NPs can modulate a range of autophagy-related (ATG) genes and signaling pathways with remarkable anti-tumor properties (Efferth and Oesch, 2021; Zhang Q. et al., 2020). In this study, we aimed to further elucidate the effect of NPs targeting autophagy on GC and its mechanism of action.

## 2 Molecular mechanism and key regulators of autophagy

**FIGURE 1:**
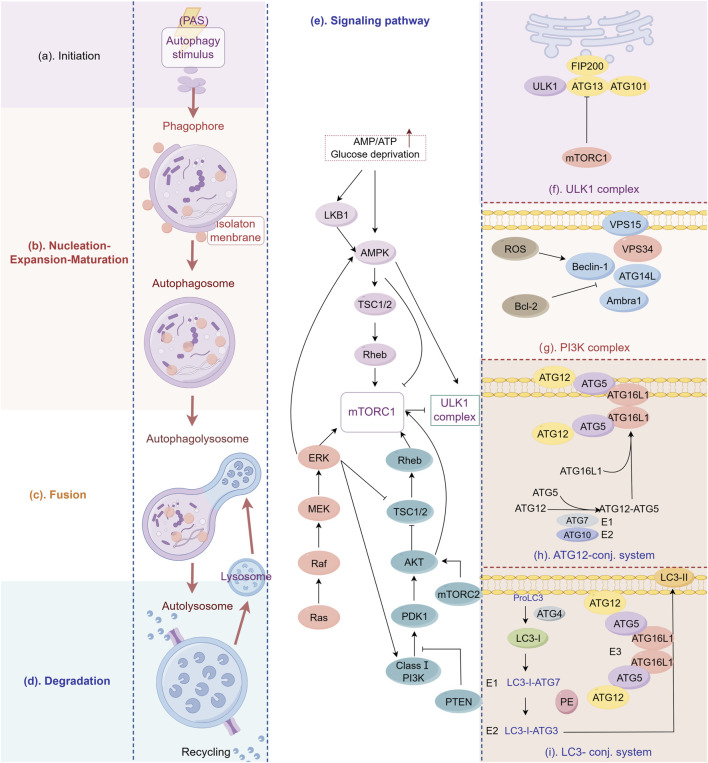
Molecular processes of autophagy and GC-related autophagy pathways. The image depicts the sequential stages and associated signaling pathways involved in autophagy, providing an intricate account of its biological progression from initiation to execution. **(A)** Initiation: Cells activate the autophagic program in response to nutrient deprivation or other stimuli. **(B)** Vesicle formation: Primary autophagosomes (isolation membranes) emerge proximally to the plasma membrane. **(C)** Expansion and maturation: The isolation membrane expands and engulfs the targeted material, culminating in fully formed autophagosomes. **(D)** Degradation: Following fusion with lysosomes, enclosed materials undergo breakdown, with degradation products subsequently recycled for reuse. **(E)** A comprehensive description of key signaling pathways regulating autophagy, including AMPK, MAPK, PI3K/AKT/mTOR, etc. **(F–I)** Showing four key protein complexes involved in autophagy regulation. The figure is drawn with Figdraw.com.

Autophagy is a complex multistep process, essentially an intracellular membrane rearrangement, which consists of the following four main phases: induction of autophagy, formation of autophagosomes (nucleation, elongation, and maturation), fusion of autophagosomes with lysosomes, and degradation of the contents in the autophagosome (Figure 1), each of which will be extensively involved and strictly regulated by a series of key factors (Li X. et al., 2020; Noda et al., 2009). The activation of autophagy mainly relies on the forward regulation of the 5'-adenylate-activated kinase (AMPK) pathway and the reverse regulation of the mammalian target of rapamycin (mTOR) pathway (Licheva et al., 2022). Once cells receive autophagy-inducing signals, a series of autophagy-related (ATG) genes are recruited to the phagocytic vesicle assembly site (PAS), where the UNC-51-like autophagy-activated kinase 1 (ULK1) complex integrates the upstream molecular signals and induces the formation of “liposome-like” double-membrane structures (Wang B. et al., 2024), also known as autophagic vesicles, which nucleate and expand to form an enveloping network to segregate their contents, with the nucleation phase relying on the formation of the coregulator Beclin-1 and the vacuolar sorting protein 34 (VPS34) (Funderburk et al., 2010). Autophagic vesicles continue to extend and expand to encapsulate cytoplasmic components (mitochondria, endoplasmic reticulum, ribosomes, etc.), and two ubiquitination coupling systems are required to participate in this phase: the first ubiquitination coupling system couples ATG5-ATG12, which in turn joins with ATG16L1 to form the ATG5-ATG12-ATG16L1 complex, which interacts with the outer membranes of extended phagocytic vesicles (Pohl and Dikic, 2019). In the second ubiquitination coupling system, microtubule-associated protein 1 light chain 3(LC3) is hydrolytically cleaved by ATG4 protein to a soluble form (LC3-I), which is subsequently coupled with phosphatidylethanolamine (PE) in response to the activation of ATG3 and ATG7, transforming it into a lipid-soluble form (LC3-II), and binds to autophagic vesicles to form autophagosomes (Sun et al., 2017). Mature autophagosomes with bilayer membranes continue to fuse with phagocytic vesicles, phagocytic vesicles, and endosomes, and their outer membranes fuse with lysosomes, and their inner membranes are degraded by lysosomes, resulting in the formation of a single-membrane autophagic lysosomal structure (Zhao et al., 2021). Lysosome-associated membrane proteins (LAMPs) protect the autophagic lysosomal membrane to ensure the degradation of the sealed contents by lysosomal hydrolases, and the degraded residues are released into the cytosol or outside the cell, whereas useful products will be recycled for cellular reuse by the action of osmolytic enzymes that mediate the transport of various molecules across the biofilm (Eskelinen, 2006). It should be noted that, as a key autophagy substrate, the expression level of p62 in cells is tightly regulated by autophagy activity, and it can negatively regulate the autophagy level of cells by activating mTORC1 (Chao et al., 2022). Therefore, The function and regulatory mechanism of p62 is an important area of autophagy and biomedical research (Zhang and Costa, 2021). The molecular processes of autophagy and GC-related autophagy pathways are illustrated in Figure 1.

## 3 Autophagy in GC

### 3.1 Autophagy related signaling pathways

The pathogenesis and progression of GC involve multiple autophagy-related signaling pathways, with the intricate signaling network formed by these pathways playing a critical role in the regulation of autophagy levels. In malignant tumors, aberrant activation of mTOR can lead to dysregulation of autophagy, which is closely linked to disruptions in tumor cell proliferation, invasion, migration, and interactions such as apoptosis and ferroptosis, ultimately accelerating GC progression (Niu et al., 2024). The following will focus on examining the mTOR pathways that are positively (PI3K/AKT/mTOR, MAPK) and negatively (p53, AMPK) regulated that are closely associated with GC, with the objective of providing insights into targeting and regulating tumor-associated autophagy signaling for optimal therapeutic outcomes. Role of key signaling pathways in GC-related autophagy is illustrated in Table 1.

**TABLE 1:** Role of key signaling pathways in GC-related autophagy.

Have access to	Molecules/complexes	Changes in autophagy levels in GC	Reference
PI3K/AKT/mTOR	PIK3CA	Stimulate AKT, increase PI3K activity, activate mTORC1 and inhibit GC autophagy, and promote tumor progression	Peng et al. (2022)
	PTEN	Up-regulating the expression of caspase-3 and p53, decreasing the ratio of Bcl-2/Bax, inducing apoptosis and autophagy death of GC cells	Glaviano et al. (2023)
	KNTC1	Inhibition of KNTC1 can induce apoptosis and autophagy of GC cells and inhibit tumor proliferation and migration	Qi et al. (2024)
AMPK	CARM1	Induction of autophagy can promote GC cell proliferation and G1-S cell cycle transformation, inhibit GC cell apoptosis, and drive cancer progression	Yang et al. (2022)
	β2-adrenergic receptor	Down-regulating the expression of p62/SQSTM1 positively regulates autophagy in GC cells and promotes the proliferation and survival of tumor cells	Zhi et al. (2019)
	Fibroblast growth factor receptor 1	Induce autophagy and mediate EMT and metastasis of GC cells	Peng et al. (2024)
	Metformin	Promote Beclin-1 dependent autophagy and inhibit proliferation, invasion and migration of GC	Liu et al. (2020a)
p53	Palbociclib	Promote lysosome formation to induce autophagy and accelerate AGS cell senescence	Valenzuela et al. (2017)
	Vitamin D3	Promote apoptosis and autophagy of AGS and SGC-7901 cells, block cell cycle, and eventually inhibit GC	Wang et al. (2023a)
	DRAM	Upregulated expression levels of LC3 and Beclin-1 induced autophagy and proliferation of SGC7901 cells	Zhu et al. (2014)
MAPK	BBR	Induced autophagy and inhibited proliferation of BGC-823 cells	Zhang et al. (2020a)
	Melatonin	By inducing ER stress to mediate apoptosis and autophagy in SGC-7901 cells, it inhibits tumor proliferation	Huang et al. (2021)

#### 3.1.1 PI3K/AKT/mTOR

The PI3K/protein kinase B (AKT)/mTOR signaling pathway is a major pathway involved in the initiation and regulation of autophagy (Barzegar Behrooz et al., 2022). As one of the most disrupted pathways in cancer, various activating mutations in oncogenes as well as inactivation of tumor suppressor genes can trigger abnormal activation of the PI3K/AKT/mTOR signaling pathway, which in turn regulates autophagy to participate in the malignant biological behaviors and prognosis of many types of tumors (Polivka and Janku, 2014). The PIK3CA gene has a significant increase in mutation frequency (14.20%) and amplification frequency (5.06%) in GC cell, and its genetic alteration can positively regulate PI3K activity to inhibit autophagy, which is closely associated with tumor progression, disease prognosis and drug resistance (Peng et al., 2022). Dysfunction or deletion of tumor suppressor phosphatase and tensin homolog (PTEN) can increase the incidence of deleterious mutations and activate AKT/mTOR signaling, thereby inhibiting autophagic death of tumor cells and promoting their proliferation and survival (Glaviano et al., 2023). In addition, elevated p-AKT levels are significantly associated with angiogenesis and tumor infiltration, and have been identified as a marker to promote GC cell migration and invasion (Matsuoka and Yashiro, 2014; Kang and Chau, 2020). A recent study revealed a new GC biomarker, Kinetochore associated 1 (KNTC1), whose high expression promotes GC cell migration, proliferation and inhibits apoptosis, and this effect is mainly associated with activation of the PI3K/AKT/mTOR pathway (Qi et al., 2024). To sum up, by targeting and regulating the PI3K/AKT/mTOR pathway, it may be an effective way to find potential diagnostic and prognostic markers for GC.

#### 3.1.2 AMPK

AMPK is a highly conserved intracellular sensor at the adenosine nucleotide level, which is mainly responsible for regulating cellular metabolism to maintain energy homeostasis (Garcia and Ampk, 2017). The sequential activation of the AMPK pathway profoundly affects the energy changes and autophagy levels of tumor cells (Wang N. et al., 2024). Studies have confirmed that Coactivator-associated arginine methyltransferase-1 (CARM1) can activate both cytoplasmic AMPK/mTOR and cytosolic AMPK/CARM1/TFE3 (Transcription factor binding to IGHM enhancer 3) signaling pathways to induce autophagy, thereby promoting GC cell proliferation and G1-S cell cycle transition, and inhibiting GC cell apoptosis in nude mice, demonstrating that the activation of AMPK/mTOR has a driving effect on GC (Yang et al., 2022). Zhi et al. first found that inhibition of adrenergic signaling could mediate the inactivation of the AMPK/ULK1 pathway, reduce autophagic flux, and ultimately inhibit GC progression and decrease tumor incidence, suggesting that the activation of the AMPK/ULK1 pathway is not only regulated by nutritional status, energy stress, growth factors, and oxygen concentration, but also has a strong link with chronic stress (Zhi et al., 2019). Peng et al. proposed that targeting stimulation of the AMPK/mTOR pathway that confers tolerance to tumor cells and induces autophagy facilitates epithelial-mesenchymal transition (EMT) and metastasis of GC cells (Peng et al., 2024). However, some investigators have proposed that downregulation of AMPK is a prerequisite for tumor cells to escape from the body’s inhibition of growth and biosynthesis (Hardie, 2011), but this downregulation mechanism can be overcome by the glucose-lowering agent metformin. Metformin activates the AMPK/mTOR signaling pathway in a dose-dependent manner, which in turn induces Beclin-1-dependent autophagy and ultimately inhibits the malignant phenotype and exerts antitumor activity in GC cells, which provides a new therapeutic idea for GC (Liu S. et al., 2020). Recent studies have proposed that AMPK can inhibit autophagy under energy-deficient conditions, which challenges the mainstream view that AMPK plays a catalytic role in the regulation of autophagy. However, the influence of AMPK pathway’s bidirectional autophagy regulation mechanism on GC cells remains to be further studied (Park et al., 2023).

#### 3.1.3 p53

As a “guardian of the genome” and a tumor suppressor, p53 gene mutation is one of the most frequent events in cancer. Mutant p53 can inhibit autophagy and exert oncogenic activity through a variety of molecular pathways, such as regulation of AMPK and AKT/mTOR pathways, hypoxia-inducible factor (HIF) (Cordani et al., 2017). Valenzuela et al. pointed that the mechanism of change in p53 regulation of autophagic flux involves the ability to regulate lysosomal protein expression, which induces autophagy by promoting lysosome formation and ultimately enhances the antiproliferative effect of Palbociclib on AGS cells (Valenzuela et al., 2017). Wang et al. demonstrated that the activation of the p53/AMPK/mTOR pathway for the promotion of autophagy and apoptosis in GC cells is a vitamin D3 exerts a dominating mechanism for anti-tumor effects (Wang Y. et al., 2023). In addition, the EMT inhibitory effect of ferritin autophagy on GC cells was significantly correlated with the activation of the p53/AKT/mTOR pathway, suggesting that p53 is a key target for preventing metastasis of GC and improving prognosis of patients (Xu Z. et al., 2020). Ogawa et al. found that the enhanced expression of p53 could induce apoptosis and autophagy in cancer-associated fibroblasts (CAFs), impede the peritoneal metastasis of GC, and improve the sensitivity of chemotherapy (Ogawa et al., 2022). Therefore, targeting p53 gene therapy may provide an effective biotherapeutic strategy for GC peritoneal metastasis. However, p53 induces protective autophagy and promotes proliferation of the SGC7901 cell line under certain circumstances (Zhu et al., 2014). Autophagy can in turn regulate p53 and may be involved in the development of resistance mechanisms to p53 activators. Inhibition of p53-induced apoptosis in tumor cells and facilitates their survival (Huo et al., 2013). In conclusion, the dual-action relationship between p53 and autophagy still needs to be further investigated to elucidate the therapeutic perspectives of these interactions in cancer.

#### 3.1.4 MAPK

The MAPK signaling pathway, also known as the RAS/RAF/MEK/ERK cascade signaling pathway, overactivation of this pathway will affect the initiation and conduct of autophagy in cancer cells, contributing to more than 40% of human cancer cases and significantly facilitating tumor metastasis and invasion (Bahar et al., 2023). In a genome-wide association study (GWAS), Wang et al. revealed that genetic variation in the MAPK pathway controls the occurrence and progression of GC in the Chinese Han population, and that there is a remarkable correlation between dysregulation of this pathway and conferring susceptibility to GC and lowering the survival of patients (Wang et al., 2020). Targeted inhibition of autophagy through modulation of the MAPK signaling pathway has become an effective strategy for the pharmacological treatment of GC. Zhang et al. found that treatment of BGC-823 cells with berberine (BBR) induced autophagy, which was manifested by increased expression of Beclin-1 and LC3-II and aberrant expression of components in the MAPK/mTOR pathway and the AKT pathway (e.g., p-mTOR, p-AKT, p-ERK, and p-p38), thus exerting its antitumor activity (Zhang Q. et al., 2020). However, the MAPK pathway can indirectly activate related bypass pathways to inhibit autophagy, such as PI3K/AKT/mTOR, which interact with each other by cross-suppressing and co-influencing the same substrate, and this complex interaction is one of the key mechanisms for the development of tolerance in cancer cells (Bou Antoun and Chioni, 2023; Saini et al., 2013). In addition, Huang et al. found that melatonin exerts anti-GC effects involves the activation of MAPK to mediate the synergistic effect between autophagy and apoptosis (Huang et al., 2021). These studies suggest that the role of MAPK-related pathways in regulating autophagy and GC progression is also two-sided, exerting anti-tumor functions by regulating multiple cell death pathways, and triggering pro-survival mechanisms to promote cell proliferation and migration.

### 3.2 Autophagy and *Helicobacter pylori* infection

**FIGURE 2:**
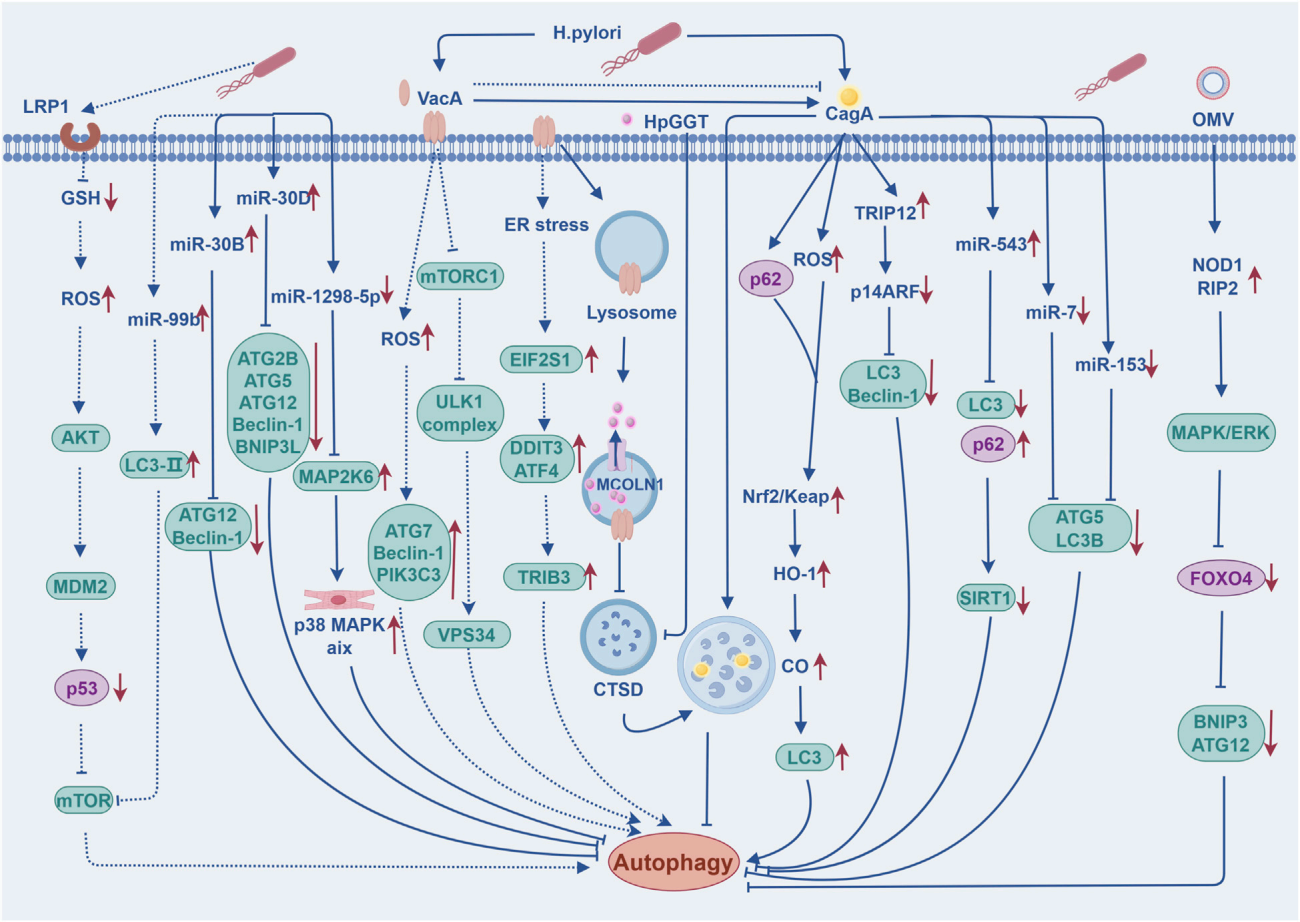
The regulatory mechanism of autophagy by *Helicobacter pylori* during acute and chronic infections. The dashed line indicates that the event occurring during the acute phase of H. pylori-I; Solid lines indicate events occurring during the chronic phase of H. pylori-I. The figure is drawn with Figdraw.com.

*Helicobacter pylori* infection (H. pylori-I) remains the most important risk factor for GC, and data from the International Agency for Research on Cancer (IARC) of the International Health Organization show that nearly 80% of new GC cases worldwide can be attributed to H. pylori-I. H. pylori-I could increase the risk of developing non-cardia gastric cancer (NCGC) and cardia gastric cancer (CGC) by more than 6-fold and 3-fold, respectively, so the eradication of *H. pylori* is of great significance for the prevention and treatment of GC (Park et al., 2018). As the duration level of cellular exposure to the toxin varies, *H. pylori* can play a dual part in the regulation of autophagy in gastric epithelial cells, either inducing or inhibiting it. These processes mainly involves two bacterial virulence factors: vacuolar cytotoxin A (VacA) and cytotoxin-associated gene A (CagA) (Wen et al., 2024). Consequently, understanding the regulatory mechanisms by which *H. pylori* modulate autophagy in GC development and progression during acute and chronic infections may be crucial for future control of the disease. The regulatory mechanism of autophagy by *H. pylori* during acute and chronic infections are illustrated in Figure 2.

#### 3.2.1 Acute infection

The induction of autophagy during acute infection serves as a crucial defense mechanism for host cells, facilitating the direct degradation of toxins, reducing cellular damage, and maintaining intracellular homeostasis (Kim et al., 2018a). Concurrently, autophagy induction is exploited by bacteria to fulfill their nutritional needs and establish an ecological niche for intracellular replication (Akbari et al., 2024). *In vitro* studies show that 12 h post-infection, H. pylori interacts with the host cell plasma membrane and subsequently internalizes into the cytoplasm within autophagosomes, thereby evading the acidic environment and immune response (Chu et al., 2010). The VacA toxin not only binds to low-density lipoprotein receptor-related protein 1 (LRP1) in gastric epithelial cells, leading to decreased glutathione (GSH) levels, reactive oxygen species (ROS) accumulation, and AKT phosphorylation, but also mediates p53 ubiquitination and inhibits the mTORC1 signaling pathway, ultimately triggering autophagic responses and the degradation of CagA (Kim et al., 2018a). Moreover, the molecular pathway of VacA-induced autophagy and apoptosis involves the suppression of integrin-linked kinase (ILK) expression (Li B. et al., 2023). ER stress significantly contributes to VacA-induced autophagic cell death by activating downstream transcriptional effectors, leading to eukaryotic translation initiation factor 2 subunit 1 (EIF2S1) phosphorylation and and increased expression of activating transcription factor 4 (ATF4), DNA-damage-inducible transcript 3 (DDIT3), and tribbles pseudokinase 3 (TRIB3), eventually inducing autophagy and apoptosiss (Zhu et al., 2017). Additionally, miR-99b can further promote H. pylori-induced autophagic response by targeting mTOR (Yang et al., 2018). The Nrf2-heme oxygenase-1 (HO-1) axis is thought to play a role in the cellular adaptive survival response occurring in H. pylori-induced GC by inducing autophagy (Paik et al., 2019). Wang et al. found that 24 h after infection, VacA activates the PINK1/Parkin pathway, mediates mitochondrial autophagy and apoptosis in GES-1 cells, and inhibits their proliferation (Wang et al., 2022). Moreover, activation of autophagy during acute infection phase is often accompanied by co-expression of a cell surface marker CD44 (Cluster of differentiation 44), which is associated with cells that induce EMT and exhibit cancer stem cell (CSC) characteristics (Courtois et al., 2021). Intracellular material recycled by autophagic degradation can also provide sufficient nutrients and energy for tumor angiogenesis (Ding et al., 2014). All these events are the result of autophagy activation by *H. pylori*, which in turn increases the risk of GC development in different ways.

#### 3.2.2 Chronic infection

The key to GC development lies in the continuous infection of *H. pylori*, during which response to autophagy inhibition will promote inflammation, evade immune surveillance, establish tumor microecology, and ultimately facilitate proliferation, metastasis and invasion of cancer cells. Continuous exposure of gastric mucosal tissues to VacA and γ-glutamyl transpeptidase (HpGGT) disrupts lysosomal calcium channels and impedes lysosomal transport, leading to the accumulation of aberrant lysosomes and autophagosomes deficient in histone D (CTSD), and ultimately generating deformed and dysfunctional vesicles that disrupt autophagosomal degradation (Capurro et al., 2019; Bravo et al., 2019). Overexpression of The AU-rich element (ARE) RNA-binding factor 1 (AUF1) can stabilize the protein levels of CagA and VacA by inhibiting lysosomal degradation, thus promoting intracellular and extracellular inflammation (Zheng H. et al., 2024). The mechanism of *H. pylori* disrupting the autophagy pathway in GC is closely related to the complex epigenetic regulatory network. For example, H. pylori-I was able to increase miR-30B expression levels in GC cells, which in turn downregulated ATG12 and Beclin-1 expression to inhibit autophagy (Tang et al., 2012). Of course, Beclin-1 downregulation can also be directly mediated by CagA (Sakatani et al., 2023). CagA could increase miR-543 expression, inhibit autophagy and promote EMT by targeting downregulation of SIRT1 (Shi et al., 2019). SIRT1 is widely recognized as a key regulator deeply involved in processes such as apoptosis, autophagy, and tumorigenesis (Patra et al., 2023). miR-30D is upregulated in response to H. pylori-I and targets downregulation of gene expression of ATG2B, Beclin-1, and BNIP3L to inhibit autophagy and promote bacterial survival (Xu et al., 2024). It has been demonstrated in chronic infection models that miR-7 in combination with miR-153 may serve as a novel diagnostic biomarker and potential therapeutic target for *H. pylori* CagA-positive associated GC. The expression levels of both decreased during H. pylori-mediated autophagic disruption, accompanied by increased GC cell proliferation and inflammatory response (Song Y. et al., 2023). p62/SQSTM1 accumulation leads to ubiquitination and degradation of DNA repair marker Rad51, which promotes mitochondrial DNA damage response and cell cycle arrest (Xie et al., 2020). In addition, as cells become tolerant to ROS, autophagy in CD44-positive GC stem cell-like cells is inhibited, favoring CagA escape autophagy and specific accumulation, which also further exacerbates damage to host cells (Tsugawa et al., 2012).

Inhibition of autophagy and exertion of pro-tumorigenic effects during the chronic infection phase of *H. pylori* are closely related to various signal transductions. Under the condition of chronic stimulation by *H. pylori* lysates, VacA and CagA delivered to host cells via outer membrane vesicles (OMVs) upregulated the expression levels of NOD1 and RIP2, and inhibited GC cell apoptosis and autophagy through the MAPK-ERK/Forkhead box subclass O protein 4 (FOXO4) pathway, ultimately inducing bacterial immune escape, tumor cell invasion and metastasis, and EMT (He et al., 2020). CagA in gastric mucosal tissues mediates the activation of the PI3K/AKT/mTOR signaling pathway, induces protease degradation of the pro-apoptotic gene Siva1, and inhibits autophagy (Palrasu et al., 2020). Li et al. confirmed that CagA protein activation of c-Met/AKT pathway negatively regulates autophagy, which can aggravate gastric mucosal atrophy and tumorigenic transformation (Li et al., 2017). Studies have confirmed that decreased expression of miR-1298-5p in H. pylori-associated GC cells leads to overexpression of its target MAP2K6, and activation of p38 MAPK signaling to inhibit autophagy has an important impact on promoting proliferation, migration and invasion of GC cells (Li X. et al., 2022). Interestingly, CagA and VacA can co-regulate autophagy and disease through a synergistic relationship. The fixed binding between the two can induce fibroblasts to differentiate in the direction of tumor, and enable gastric epithelial cells to transform into EMT (Noto et al., 2019). The persistence of CagA also depends on the inhibition of autophagy by VacA (Abdullah et al., 2019). In recent years, some researchers have proposed that the regulation of autophagy signaling by *H. pylori* causes changes in the structure of the gut microbiota, which may provide the basic conditions for the occurrence and progression of GC (Nabavi-rad et al., 2023). In conclusion, targeting autophagy under different infection periods of *H. pylori* may be an important direction to intervene in gastric mucosal malignancies.

### 3.3 Autophagy and other cell death

Autophagy drives different modes of death and serves as a basis for initiating other modes of death (Galluzzi et al., 2018). Among them, the crosstalk between autophagy and apoptosis and ferroptosis has attracted much attention in the context of GC, but still has not been fully and accurately demonstrated (Wen et al., 2024; Hu C. et al., 2023). The following section details the mechanisms by which autophagy interacts with apoptosis and ferroptosis and how it affects GC.

#### 3.3.1 Crosstalk between autophagy and apoptosis in GC

Autophagy and apoptosis serve as protective mechanisms to mitigate cellular damage under stress conditions. Typically, autophagy precedes apoptosis and acts to inhibit it; however, apoptosis may be triggered if the stress stimulus surpasses a critical threshold. The interplay between these processes is contingent upon the nature of the stress stimulus, as well as its intensity and duration (Biswas et al., 2024). In turn, apoptotic signals and apoptotic products can also promote or inhibit autophagy (Djavaheri-Mergny et al., 2010). Apoptosis is a critical process in the suppression of tumor formation and tumor cells can respond to adverse microenvironments by inducing protective autophagy and inhibiting apoptosis (Sun et al., 2011). Endoplasmic reticulum (ER) stress plays an important role in the antagonistic relationship between autophagy and apoptosis, and is ultimately involved in the complex mechanisms that influence tumour progression and treatment resistance (Liao et al., 2025). For example, tretinoin lactone may contribute to ROS accumulation in GC cells, whereas ROS-induced ER stress activates both apoptosis and autophagy, and increased autophagic flux has an anti-apoptotic effect. Therefore, the combination of tretinoin and autophagy inhibitors may achieve more desirable results in the clinical treatment of GC (Chen P. et al., 2024). Gao et al. found that the acidic microenvironment induced autophagy in GC cells by activating the MAPK/ERK1/2 pathway, while the expression of the pro-apoptotic protein Bax was decreased and the expression of the anti-apoptotic proteins Bcl-2 and NF-κB was increased compared to the neutral condition, suggesting that autophagy may attenuate the susceptibility of GC cells to apoptosis and may help explain why the acidic microenvironment is more conducive to aggressive growth and metastasis of tumour cells (Gao Y. et al., 2019). Not only that, apoptosis-associated caspase activation also inhibits the autophagy process, and the imbalance between apoptosis and autophagy is accompanied by excessive proliferation of tumour cells. Zhang et al. revealed for the first time that the megakaryoblastic leukemia type 1 (MKL1)/miR-5100/caspase activity and apoptosis inhibitor 1 (CAAP1) formed a new regulatory loop for GC cell growth. miR-5100 promotes apoptosis and inhibits autophagy in GC cells, CAAP1 inhibits apoptosis and promotes autophagy in tumor cells, and at the same time, MKL1 can target the miR-5100 promoter and promote the expression of miR-5100, and the three of them regulate the apoptosis and autophagy of normal cells together. Once external stimuli cause MKL1 and miR-5100 to be difficult to counterbalance each other, cellular autophagy and apoptosis will be induced to become disordered and cancerous (Zhang H. M. et al., 2020).

However, autophagy does not simply serve as a survival mechanism for tumor cells to help themselves evade apoptosis. circRELL1, a novel GC-associated cyclic RNA, is involved in autophagy activation and negatively regulates miR-637 has been shown to reverse the malignant phenotypes of proliferation, resistance to apoptosis, and migration of tumor cells (Sang et al., 2022). Cai et al. proposed for the first time that argininosuccinate synthase 1 (ASS1) exerts its oncogenic potential by a mechanism of action related to the inhibition of lysosomal degradation of LC3-II in intrinsic autophagosomes and protection of cancer cells from chemotherapeutic drug-induced apoptosis (Tsai et al., 2018). Apparently, at this point, autophagy mediates not a protective effect on tumor cells but induces apoptosis and synergistically promotes tumor cell death. The synergistic effect between autophagy and apoptosis is also reflected in the anti-GC activity of naringin. It can block SNU-1 cell proliferation and cell cycle arrest, and induce SNU-1 cell apoptosis by inhibiting PI3K/AKT signalling and activating autophagy. Resistance to tumour cell immortalisation by multiple mechanisms demonstrates the potential value of naringin as an anti-GC agent (Xu C. et al., 2021). In conclusion, clarifying the mode of interaction between autophagy and apoptosis is an prominent prerequisite for the treatment of GC.

#### 3.3.2 Interplay between autophagy and ferroptosis in GC

Ferroptosis, is a unique form of cell death with three key mechanisms: GSH biosynthesis, lipid peroxidation and iron metabolism (Deng et al., 2023). Many types of autophagy are involved in regulating ferroptosis, and the key molecules involved in ferritin, such as ferritin and lipids, can also be used as substrates for autophagy (Zhou et al., 2020). The complex cyclic network between autophagy and ferroptosis processes relies on oxidative stress, and the synergistic regulation of them will lead to a great therapeutic potential for tumours.

In GC, autophagy is widely acknowledged to enhance ROS-dependent ferroptosis. It facilitates lipid peroxidation and oxidative stress by degrading ferritin and inducing the expression of transferrin receptor 1 (TfR1), which subsequently promotes ferroptosis in tumor cells (Hou et al., 2016; Park and Chung, 2019). The specific characteristics of each autophagy-related gene, quantified as an autophagy score, determine its differential capacity to regulate ferroptosis. Li et al. categorized the identified autophagy-related genes crucial for GC prognosis into three distinct autophagy modes and observed that the expression of ferroptosis-related genes was positively correlated across these modes, despite some variations. Autophagy inhibitors corresponding to these three modes were capable of reversing the reduction in cell viability and lipid accumulation induced by erastin, a ferroptosis inducer, to varying extents. This finding underscores the significance of the autophagy score in predicting the sensitivity of GC patients to ferroptosis-based treatments and in assessing patient prognosis (Li H. et al., 2022). Shang et al. initially demonstrated that the overexpression of circHIPK3 inhibited autophagy and ferroptosis, as indicated by a marked reduction in the expression levels of autophagy-related markers such as LC3-II and Beclin-1. This was accompanied by a decrease in ROS generation, GSH depletion, and the accumulation of redox-active iron (Fe2+) in GC cells. Notably, the activation of autophagy was capable of reversing the inhibition of ferroptosis induced by circHIPK3 overexpression, and significantly diminished cisplatin (DDP) resistance in GC cells (Shang et al., 2023). Furthermore, ATG genes or pathways influence the sensitivity of tumor cells to ferroptosis. For instance, cancer cells harboring mutations in the PIK3CA gene or deletions in PTEN exhibited oncogenic activation of the PI3K/AKT/mTORC1 pathway, resulting in reduced sensitivity to lipid peroxidation and resistance to ferroptosis. The combination of mTORC1 inhibitors with ferroptosis inducers significantly decreased tumor size (Yi et al., 2020). Interestingly, certain ATG genes can respond to alterations in cellular metabolism and environmental conditions, thereby exerting dual regulatory effects on autophagy and ferroptosis. In normal cells, the protein p53 facilitates both autophagy and ferroptosis in response to cellular stress. Conversely, in cancer cells harboring p53 mutations, the loss of p53 functionality results in diminished autophagy and heightened resistance to ferroptosis, thereby providing a selective advantage for tumor cells (Corazzari and Collavin, 2023). Recently, a novel regulatory pathway of autophagy-induced ferroptosis, the AKT/glycogen synthase kinase 3 beta (GSK-3β) pathway, has been identified. Mitochondrial glutathione S-transferase 1 (MGST1) can activate this pathway, leading to the inhibition of ATG16L1 expression and the conversion of LC3-I to LC3-II, and subsequently preventing the degradation of autophagic ferritin, and ultimately results in GC cell resistance to ferroptosis (Peng and Peng, 2023).

## 4 Natural products regulate GC through autophagy

**FIGURE 3:**
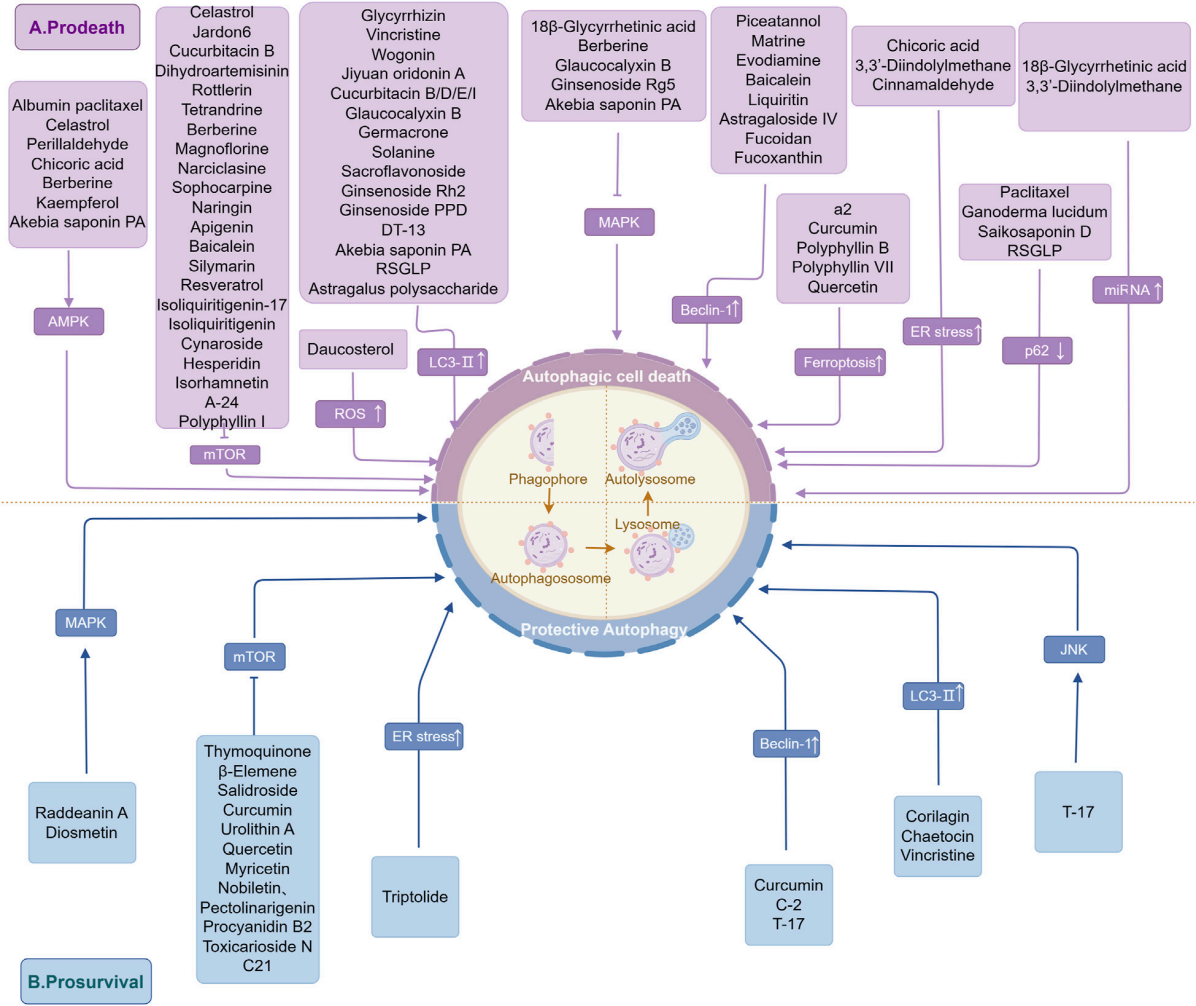
The underlying mechanism of NPs targeting autophagy for intervention in GC. This figure summarizes the results of current studies on the mechanism of regulation of GC autophagy by NPs and demonstrates the interaction of NPs with GC-associated ATGs and signaling pathways. These include, **(A)** NPs that induce autophagic cell death to exert an anti-GC effect; **(B)** NPs that induce protective autophagy in favor of tumor survival. The figure is drawn with Figdraw.com.

Currently, most of the modern clinical antitumor drugs are chemically synthesized, which often have the problems of single target point, long treatment period, high side effects, and drug dependence (Macdonald, 2006; Gao et al., 2017). Benefiting from thousands of years of extensive clinical applications and rich experience, TCM occupies a unique advantage in GC treatment. Modern pharmacological studies have unearthed numerous NPs with Anti-tumor activitity related to the regulation of autophagy, linking ATG genes, proteins, and signaling pathways to NPs and exerting autophagy-inducing/inhibitory effects, which ultimately inhibit tumor cell growth, metastasis, and improve chemosensitivity (Liu et al., 2022). Terpenoids, alkaloids, and flavonoids are all anticancer adjuvants widely used clinically to enhance therapeutic efficacy and mitigate side effects (Chen M. et al., 2024; Xu et al., 2018; Wu et al., 2018). The following section summarizes the NPs and potential mechanisms that regulate autophagy in GC cells, with the aim of helping in the treatment of cancer and drug development. The underlying mechanism of NPs targeting autophagy for intervention in GC is illustrated in Figure 3.

### 4.1 Terpenoids

18β-Glycyrrhetinic acid (18β-GRA) is an active metabolite of Glycyrrhizic acid derived from licorice root, which exhibits a wide range of pharmacological effects including anti-inflammatory, antioxidant, anticancer, and hepatoprotective activities (Shinu et al., 2023). Research has found that 18β-GRA can activate the miR-345-5p/TGM2 (tissue transglutaminase 2) signaling pathway, regulate the expression of autophagy markers (ULK1, p62, LC3I/II), increase autophagic flux, ultimately inhibiting tumor cell viability, promoting apoptosis in GC cells and blocking their proliferation (Li et al., 2023b). Shortly after, Yang et al. discovered that the miR-328-3p/STAT3 signaling pathway is closely associated with the anti-gastric cancer activity of 18β-GRA (Yang et al., 2023). Overactivation or aberrant expression of components in the MAPK signaling pathway can enhance cell toxicity and induce protective autophagy in cancer cells, which becomes a critical factor limiting clinical efficacy (Miao et al., 2024). Xia and colleagues employed network pharmacology and experimental validation to unveil that 18β-GRA can decrease the miRNA and protein expression levels of KRAS, ERK1, and ERK2 in AGS cells, targeting inhibition of the MAPK signaling pathway for its anti-tumor effect (Li et al., 2023c). Additionally, Glycyrrhizic acid can reverse H. pylori-induced damage to the autophagy-lysosomal pathway in AGS cells and restore lysosomal activity, inducing autophagy while inhibiting *H. pylori* growth. It may serve as an autophagy activator providing new strategies for GC treatment in the future (Khan et al., 2023). Paclitaxel (PTX) is a diterpenoid alkaloid compound that has gained worldwide attention as an anti-cancer star and research focus due to its high efficiency, low toxicity, and broad spectrum advantages (Pei et al., 2023). Previous studies have shown that PTX can trigger autophagy inhibition of GC cell proliferation (Yu et al., 2017). Laterly, it was discovered that PTX-induced protective autophagy plays a major role in chemotherapy resistance, leading to increased interest in combination therapy with PTX (Škubník et al., 2023). The nanoscale albumin-bound paclitaxel formulation developed by Celgene Corporation in the United States (Nab-PTX) demonstrates superior anti-tumor effects in GC compared to commonly used anti-tumor drugs 5-fluorouracil (5-FU) and lobaplatin (LBP), with significant disparities observed in its ability to induce autophagy and cell apoptosis (Cheng et al., 2022). When PTX is combined with oncolytic adenovirus OBP-401 which has lytic activity, it can induce mitotic catastrophe in GC cells, downregulate p62 expression and promote PARP accumulation, accelerating autophagy and cell apoptosis while enhancing the oncolytic effect of the virus. Both treatments synergistically inhibit tumor cell proliferation and metastasis (Ishikawa et al., 2020; Khing et al., 2021). Additionally, Yu et al. proposed that autophagy mediates the superior anticancer effects and fewer side effects of paclitaxel nanoparticles (PTX-NPs) through ROS response (Yu et al., 2019). Interestingly, research has revealed that the anticancer effect of PXT can be further potentiated by Cycloastragenol; however, its primary mechanism lies in promoting cell apoptosis rather than autophagy (Hwang et al., 2019).

Ganoderma lucidum, renowned as one of Chinese precious medicinal fungi, has been scientifically proven to possess immunomodulatory, antihypertensive, anti-tumor, hepatoprotective and liver-protective properties (Liu et al., 2023). Currently, the key constituents of Ganoderma lucidum targeting cancer cells are triterpenoids extracted using methanol/ethanol solvents (Liu X. et al., 2020). Treatment of tumor cells with methanol extract from Ganoderma lucidum can stimulate autophagosome formation and elicit similar effects to autophagy induction (evidenced by increased LC3-II levels and decreased p62 levels), thereby demonstrating its potential as an autophagy inducer for anti-tumor activity (Oliveira et al., 2014). Celastrol is a pentacyclic triterpenoid compound exhibiting broad-spectrum anticancer activity derived from Tripterygium wilfordii Hook F. roots (Wang C. et al., 2023). Li et al. discovered that celastrol activates the AMPK pathway while deactivating the AKT/mTOR signaling pathway, inducing both autophagy and cell apoptosis, finally leading to inhibition of GC cell proliferation and reduction in cellular burden (Lee et al., 2014). Jiyuan oridonin A (JDA), a kaurene diterpenoid derived from Rabdosia rubescens, exhibits time and dose-dependent effects on GC cells. At low doses, JDA induces tumor cell senescence by inactivating the c-Myc-AP4 pathway, while high doses of JDA upregulate the expression levels of LC3B, cleaved PARP, and cleaved caspase-3 in GC cells. In essence, JDA activates autophagy to induce cell apoptosis, offering an effective treatment for GC based on disease progression (Wang Y. et al., 2021). Additionally, Liu and his team first demonstrated that the specific mechanism by which the JDA derivative a2 exerts its anti-GC activity is closely related to ferroptosis mediated by iron accumulation through autophagy pathways (Liu et al., 2021). Jaridon 6, a novel diterpenoid compound extracted from Rabdosia rubescens, possesses advantages in treating drug-resistant GC as it inhibits the PI3K/AKT pathway to suppress SIRT1 enzyme activity and induce compensatory autophagic cell death in resistant tumor cells as compensation for resistance to apoptosis of tumor cells (Fu L. et al., 2021). Cucurbitacin B (CuB) is a tetracyclic triterpenoid compound derived from the Cucurbitaceae family, exhibiting broad pharmacological activities (Dai et al., 2023). Cancerous inhibitor of protein phosphatase 2A (CIP2A), a human cancer protein, exerts carcinogenic activity by activating and inhibiting downstream effectors based on protein phosphatase 2A (PP2A) (Khanna et al., 2013). Liu et al. discovered that CuB can suppress proliferation, induce caspase-dependent apoptosis and autophagy in SGC7901 cells by inhibiting the CIP2A/PP2A/mTORC1 signaling axis, providing a potential candidate drug for overcoming cisplatin resistance (Liu et al., 2017). Apart from CuB, CuD, CuE, and CuI have been shown to induce GC cell death by upregulating the expression levels of autophagy-related marker LC3; among them, CuI exhibits the strongest anti-GC potency (Jafargholizadeh et al., 2018). Perillaldehyde (PAH), an active ingredient isolated from traditional herb Perilla frutescens var. crispa, effectively inhibits tumor cells proliferation and induces apoptosis in mouse GC cell line MFCs xenograft model through promoting AMPK phosphorylation and activation of autophagy (Zhang et al., 2019). Furthermore, Glaucocalyxin B (GLB), a natural compound isolated from aerial parts of Rabdosia japonica, significantly upregulates LC3A/B-II expression and activates caspases while regulating Bcl-2 family proteins expression. It ultimately enhances sensitivity of GC cells to chemotherapy drugs while reducing drug side effects through multiple pathways including blocking cell cycle progression, impairing DNA replication as well as promoting apoptosis and autophagy (Ur Rahman et al., 2017). In previous studies, Germacrone (GM), a sesquiterpene compound extracted from Curcuma zedoaria, has been identified for its diverse pharmacological activities including anti-tumor, anti-inflammatory, antitussive, and vasodilatory effects (Liu et al., 2013; Fang et al., 2023). Fang et al. proposed that GM can reverse the proliferation of GC cells induced by overexpression of hepatitis B X-interacting protein (a broad-spectrum carcinogenic protein). It inhibits cell apoptosis and autophagy by promoting autophagosome formation through an increase in LC3-II/LC3-I ratio (Fang et al., 2020). Dihydroartemisinin (DHA), derived from Artemisia annua L. and widely used for malaria treatment, also exhibits significant anticancer activity. It enhances cellular autophagy by inhibiting the PI3K/AKT/mTOR pathway and induces cell apoptosis via caspase-dependent and mitochondrial pathways. Additionally, it increases sensitivity to DDP by suppressing P-glycoprotein expression (a multidrug resistance membrane protein) (Eichhorn and Efferth, 2012; Zhang et al., 2022).

However, several NPs such as Thymoquinone, β-Elemene, and Salidroside have been reported to exhibit a close correlation between their anti-gastric cancer properties and the inhibition of cell apoptosis mediated by the PI3K/AKT/mTOR signaling pathway (He et al., 2023; Liu et al., 2011; Rong et al., 2020). A recent study has demonstrated that Triptolide induces a cascade effect in gastric cancer cells, initially triggering ROS accumulation and subsequently inducing protective autophagy in response. This ultimately leads to its cytotoxic effects. Simultaneously, ROS accumulation also contributes to Triptolide-induced cell apoptosis, which may play a crucial role in its anticancer properties. Therefore, combining autophagy inhibitors with Triptolide could potentially synergistically enhance anticancer efficacy while reducing side effects (Chen P. et al., 2024). Raddeanin A (RA), a natural triterpenoid compound derived from Anemone raddeana regel, can activate the p38 MAPK pathway to induce apoptosis and autophagy in SGC-7901 cells. Combining RA with autophagy inhibitors can augment anticancer efficacy (Teng et al., 2016). In conclusion, numerous natural triterpenoid compounds hold promise as high-quality potential anticancer drugs through modulation of relevant molecules and signaling pathways involved in autophagy and cell apoptosis.

### 4.2 Polyphenols

Curcumin (CUR), a natural polyphenolic antioxidant primarily derived from Curcuma longa, exhibits lipid-lowering, anti-tumor, choleretic, and antioxidant effects (Hu P. et al., 2023; Fu YS. et al., 2021). In GC cells, CUR inhibits the PI3K/AKT/mTOR pathway while activating the p53 signaling pathway. Furthermore, it upregulates the expression levels of Beclin-1, ATG3, and ATG5 and promotes dynamic conversion from LC3-I to LC3-II (Fu et al., 2018). Xiao et al. also suggested that CUR demonstrates dose-dependent inhibition of autophagy and cell apoptosis induced by the PI3K/AKT/mTOR pathway to exert its anti-GC effect in conjunction with a ketogenic diet (Xiao et al., 2022). Moreover, recent studies have confirmed that targeting inactive PI3K/AKT/mTOR pathways with CUR can induce autophagy-mediated ferroptosis, characterizing by increased levels of Fe2+, MDA, lipid ROS as well as acyl-CoA synthetase long-chain family member 4 (ACSL4), a key biological marker for ferroptosis induction; meanwhile suppressing GSH expression levels along with SLC7A11 and GPX4 in GC cells, ultimately leading to suppression of GC cell growth (Zheng X. et al., 2024). Rottlerin, a natural polyphenol extracted from Mallotus philippinensis, has been shown by Song et al. to induce autophagy in a dose-dependent manner through the inhibition of mTOR and S-phase kinase-associated protein 2 expression, thereby mediating apoptosis in SGC-7901 and MGC-803 GC cell lines. Notably, this anticancer mechanism is independent of caspase (Song et al., 2018). Piceatannol (PIC) is a natural polyphenolic stilbene compound that has been recognized for its superior anti-inflammatory, antioxidant, and antiproliferative activities. The binding and phosphorylation of Bcl-2 with Beclin-1 are crucial bridges between autophagy and apoptosis (Prerna and Dubey, 2022). PIC can inhibit the phosphorylation activity of Beclin-1 and hinder its binding with Bcl-2, further triggering Beclin-1-dependent autophagic signaling activation, effectively suppressing tumor progression in GC xenograft models and inducing cell apoptosis. It synergizes with the mTOR inhibitor to achieve anticancer purposes (Huangfu et al., 2023). Chicoric acid (CA), a natural compound mainly derived from chicory and purple coneflower, can induce autophagy by promoting AMPK pathway activation and ER stress. This is manifested by upregulation of Beclin-1, ATG5, LC3-II expression, while p62 expression decreases, proving the important potential of CA in preventing and treating GC (Sun et al., 2019). Signorelli et al. demonstrated that Resveratrol induces autophagy in GC cells, leading to cell death during the early stages of cancer progression. This mechanism of action may be attributed to Resveratrol’s inhibition of dihydroceramide desaturase and subsequent disruption of the balance between dihydroceramides and ceramides accumulation (Signorelli et al., 2009). It has been established that the ratio of dihydroceramides/ceramides plays a crucial role in determining the cytotoxicity and autophagic response in cancer cells, with an elevated ratio triggering ER stress and consequent activation of autophagy through upregulation of TRIB3-mediated AKT-mTORC1 axis inhibition (Hernández-Tiedra et al., 2016).

Autophagy is often a double-edged sword in the fight against cancer, therefore accurately identifying different types of tumor cells and the ‘threshold’ between autophagy-mediated cell death and survival at each stage of development will be a prerequisite for targeted autophagy therapy. Some studies have yielded contradictory conclusions, demonstrating that certain NPs-mediated autophagy is beneficial to GC cell survival. Urolithin A, a metabolite produced in the intestines from foods rich in ellagitannins such as pomegranates and walnuts, promotes LC3-II accumulation and p62 degradation by downregulating the PI3K/AKT/mTOR pathway, increasing expression of apoptotic protein Bax while decreasing expression of anti-apoptotic protein Bcl-2. However, compared to treatment with Urolithin A alone, when used in combination with an autophagy inhibitor, the proportion of apoptotic cells significantly increases and further regulates levels of apoptosis-related proteins Bax and Bcl-2. This suggests that Urolithin A mediates protective autophagy and cell apoptosis through the PI3K/AKT/mTOR pathway induction, ultimately inhibiting tumor progression and reducing chemotherapy drug toxicity (Zhang Y. et al., 2023). Corilagin, a polyphenolic tannic acid compound derived from Phyllanthus niruri L., has been shown that can induce caspase-dependent cell apoptosis and autophagy in GC. This is evidenced by the activation of caspase-8/9/3 and PARP proteins, as well as the promotion of LC3 conversion from LC3-I to LC3-II. Additionally, it facilitates ROS accumulation leading to reduced cell viability and inhibition of proliferation in GC cells. However, the autophagy induced by Corilagin can counteract the detrimental effects on cancer cell survival caused by apoptosis induction and growth suppression (Xu et al., 2019). In summary, these studies suggest that polyphenolic compounds serve as regulators of autophagy induction/inhibition, providing valuable insights for GC prevention and treatment.

### 4.3 Alkaloids

The complex molecular patterns and signal transduction processes in tumor progression can be influenced by alkaloids. Extensive research has demonstrated the significant potential of naturally occurring alkaloid compounds in the chemical prevention and treatment of cancer. For instance, 3,3′-Diindolylmethane (DIM), a major bioactive indole derived from cruciferous plants, exhibits therapeutic effects against various gastrointestinal diseases and possesses anti-cancer properties (Kim, 2016; Thomson et al., 2016). It was found that autophagy occupies an important position in the anti-tumor activity of DIM, but the mechanism is not to induce autophagy activation, but to reverse the inhibitory effect of ATG5 by reducing the expression of miR-30e in BGC-823 cells, and to inhibit the proliferation of tumor cells by interfering with autophagosome degradation, indicating that DIM responds to the autophagic death of cancer cells by its cytotoxic effect (Ye et al., 2016). Dysregulation of Ca2+ homeostasis is extensively implicated in tumor cell proliferation, cell death, immune response, and other processes, emerging as a pivotal driving force in cancer progression (Zheng et al., 2023). Yang et al. proposed an alternative regulatory mechanism underlying DIM-induced GC cell death and revealed that DIM treatment induced cytoplasmic Ca2+ overload in human BGC-823 and SGC-7901 cells. The resultant accumulation of free Ca2+ within the cytoplasm contributed to p-AMPK-mediated ER stress, finally triggering apoptosis and autophagy in cancer cells (Ye et al., 2021). Tetrandrine (TET), a bisbenzylisoquinoline alkaloid extracted from Stephania tetrandra S., has been shown to have various pharmacological activities including anti-tumor effects (Chen, 2002). In GC, TET promotes autophagy and cell apoptosis of HGC-27 cells by inhibiting activation of the PI3K/AKT signaling pathway and upregulating expression of autophagy-related markers (Beclin-1, LC3-II, and p62) as well as cleavage of apoptosis-related markers (caspase-3,caspase-9,and PARP) (Bai et al., 2018). Matrine isolated from the roots and stems of traditional Chinese herb Sophora flavescens supports evidence for matrine-triggered autophagy. Zhang et al.'s study demonstrated that matrine significantly upregulated expression of autophagy marker Beclin-1 and pro-apoptotic protein Bax in human GC cells, providing an effective therapeutic strategy for inhibiting GC development (Zhang et al., 2011). Similarly, Rasul et al. demonstrated that Evodiamine synergistically induces autophagy and apoptosis to exert anti-tumor activity. Specifically, Evodiamine activates autophagy and apoptosis in a dose-dependent manner in GC cells. The induction of autophagy is associated with upregulation of Beclin-1 expression, while the pro-apoptotic mechanism involves regulation of Bcl-2 and Bax expression (Rasul et al., 2012). Berberine(BBR), the main bioactive component of Rhizoma Coptis traditionally used for treating inflammatory diseases and metabolic disorders such as diabetes (Haftcheshmeh et al., 2022; Xu X. et al., 2021), exhibits targeted modulation on multiple steps and phenotypes in Correa cascade for preventing and treating GC based on emerging preclinical evidence. In addition to inhibiting MAPK pathway and P13K/AKT/mTOR pathway to suppress GC cell proliferation, induce autophagy and apoptosis, BBR also inhibits AMPK pathway and NF-κB signaling pathway to reduce H. pylori-induced gastric mucosal inflammation, delaying or even reversing “inflammation-cancer” transformation. Furthermore, it regulates AMPK pathway and TGF-β1 (Transforming growth factor-β1)/Smad pathway to inhibit invasion and migration of GC cells (Liu et al., 2022). In conclusion, due to its advantages such as multi-targeting effects on multiple pathways, etc., BBR holds great potential application value in the prevention and treatment of GC. Magnoflorine induces autophagy and cell apoptosis in GC by activating ROS expression, thereby exerting an anti-tumor effect and reducing the volume of GC tumors. This effect is primarily mediated through inhibition of AKT signal transduction and downregulation of mTOR phosphorylation levels, leading to increased expression levels of LAMP1, p62, and LC3B-II. Additionally, it activates the JNK pathway to enhance PARP, caspase-3, and Bax cleavage while suppressing Bcl-2 expression (Sun et al., 2020). In addition, narciclasine has been reported to induce autophagy-dependent cell apoptosis in GC cells through modulation of AKT/mTOR phosphorylation (Yuan et al., 2021). The bioactive substance sophocarpine extracted from Sophorae flavescentis promotes GC cell apoptosis and autophagy by regulating the PI3K/AKT signaling pathway (Huang et al., 2019). Recently, Tang et al. proposed a novel pathway for inhibiting tumor growth through solanine (an extract from traditional Chinese herb Solanum nigrum Linn), which involves inhibition of autophagy via Adipogenesis associated Mth938 domain containing (AAMDC) signaling pathway activation. Previous studies have shown that AAMDC activates PI3K/AKT/mTORC1 signal transduction to promote malignant proliferation of breast cancer cells (Golden et al., 2021). Overexpression of AAMDC negatively affects GC cell proliferation inhibition and cell apoptosis, but Solanine can activate autophagy (promote LC3 conversion) and induce cell apoptosis by modulating AAMDC-related pathways in both GC mouse models and cellular models to prevent GC occurrence and inhibit tumor progression, thus confirming the potential of Solanine as a natural autophagy inhibitor for treating GC (Tang et al., 2023).

It is worth noting that as the progression stage of tumors changes, the cytotoxic effect of some NPs-mediated autophagy on malignant cells may be replaced by a protective effect. For example, GC cells continuously exposed to vincristine will release High-mobility group box 1 (HMGB1) into the extracellular space through autophagy induction and upregulate the expression level of myeloid cell leukemia-1 (MCL-1), which is a critical anti-apoptotic member of the Bcl-2 protein family. The degradation of MCL-1 is crucial for tumor cell apoptosis and sensitivity to chemotherapy drugs (Bolomsky et al., 2020). Therefore, autophagy significantly counteracts vincristine-induced cell apoptosis and provides an advantageous environment for tumor survival (Zhan et al., 2012). Chaetocin derived from fungi in the genus Chaetomium has been shown by Liao et al. to provide strong evidence that autophagy induced by Chaetocin promotes GC cell escape from death. After treating GC cells with Chaetocin, a significant upregulation in LC3B-II expression level was observed while p62 showed opposite effects. However, treatment with chloroquine resulted in reduced autophagic flux and significantly decreased survival ability, indicating that combining Chaetocin with autophagy inhibitors helps promote cancer cell death (Liao et al., 2019). In summary, whether mediating pro-survival/pro-death mechanisms in tumors, these alkaloids provide solid evidence for developing new therapeutic strategies for GC.

### 4.4 Flavonoids

Flavonoid compounds are commonly used therapeutic drugs for preventing and treating cancer due to their broad range of biological activities such as inhibiting proliferation, inflammation, invasion, metastasis, activating autophagy and inducing cell apoptosis (Kopustinskiene et al., 2020). Naringin (Nar) is a dihydroflavonoid compound found abundantly in various medicinal plants and fruits. Raha et al. attributed the inhibitory effect of Nar on tumor growth to its ability to activate ERK1/2,JNK,and p38 MAPK members within the MAPK family which then suppresses the PI3K/AKT/mTOR cascade reaction resulting in autophagy-related growth inhibition specifically targeting GC cells (Raha et al., 2015). Subsequently, they further discovered that Nar-induced autophagic cell death involves two aspects: On one hand, it involves LAMP1-mediated lysosomal damage, leading to lysosome-dependent cell death in AGS cancer cells. On the other hand, Nar activates BH3 proteins in AGS cells, triggering cell death through the propagation of intrinsic apoptotic signaling pathways (Raha et al., 2020; Cerella et al., 2020). Research has confirmed that apoptosis also participates in Nar’s therapeutic effect on GC. Specifically, Nar blocks the PI3K/AKT signaling pathway in SNU-1 GC cell lines, significantly inducing autophagy and increasing the expression of LC3B-II and Beclin-1 while downregulating p62 expression, thereby inducing apoptosis in SNU-1 cells (Xu C. et al., 2021). Additionally, when Naringin or Naringenin is combined with other targeted anticancer drugs for administration, they can synergistically enhance the anticancer efficacy of the targeted drug (Ganesh et al., 2023). It has long been established that disruption of oxygen homeostasis is closely associated with the biological characteristics and malignant phenotypes of cancer cells. Cancer cells create a hypoxic tumor microenvironment for their own survival where hypoxia-inducible factor 1α (HIF-1α) plays a pivotal regulatory role in metabolic reprogramming observed in hypoxic cancer cells (Chen et al., 2023). Enhancer of zeste homolog 2 (Ezh2) overexpression has been shown to be involved in several key events such as GC development, infiltration, metastasis, and drug resistance (Yu et al., 2023). Jin et al. demonstrated that Apigenin (APG) inhibits HIF-1α and Ezh2 by suppressing mTOR signaling in GC and induces ER stress and autophagy-related cell death through the protein kinase RNA-like ER kinase-activating factor 4-C/EBP homologous protein (PERK-ATF4-CHOP) pathway in GC cells (Kim and Lee, 2021). APG treatment enhances autophagic flux in AGS cells by inhibiting the PI3K/AKT/mTOR pathway and upregulating the expression levels of LC3B-II and Beclin-1. However, unlike Nar, APG significantly increases p62 protein levels, which appears contradictory to the notion that p62 negatively regulates autophagic activity (Kim SM. et al., 2020). Potential therapeutic effects of Baicalein have garnered considerable attention across various cancer types. Li et al. proposed that Baicalein can serve as a sensitizer for DDP chemotherapy drugs due to its ability to induce apoptosis and autophagy in GC cells by modulating AKT/mTOR and oxidative stress-related Nrf2/kidney injury molecule-1 (Keap-1) pathways (Li P. et al., 2020). Furthermore, Wogonin derived from traditional Chinese herb Scutellaria baicalensis has been combined with the anticancer drug Oxaliplatin to enhance cytotoxicity while minimizing side effects. This combination facilitates dose reduction while achieving optimal therapeutic outcomes through increased LC3-II expression levels and ULK1 phosphorylation-induced autophagy as well as induction of nitrosative stress, promotion of JNK phosphorylation, and reduction of mitochondrial membrane potential leading to apoptosis induction in BGC-823 cells (Hong et al., 2018).

Silymarin, extracted from Silybum marianum L., is commonly used for hepatoprotection treatment. Increasing research has found its significant anticancer effects in GC. silymarin-functionalized selenium nanoparticles (Si-SeNPs) induce autophagy and exert cytotoxicity by inhibiting the PI3K/AKT/mTOR pathway, leading to apoptosis and ultimately promoting cancer cell death (Mi et al., 2022). The bioactive compound Cinnamaldehyde derived from Cinnamomum cassia exhibits significant anti-cancer activity. Studies have demonstrated that Cinnamaldehyde modulates the PERK-CHOP signaling pathway to enhance intracellular Ca2+ release in GC cells and counteracts the inhibitory effect of histone lysine N-methyltransferase G9a binding with ATGs (such as LC3B, Beclin-1, HIF-1) on autophagy. Consequently, it induces ER stress and triggers autophagic cell death (Kim, 2022). Similarly, Kaempferol, a natural flavonoid widely distributed in vegetables, fruits and traditional medicinal plants, has been found to induce autophagic cell death in GC cells through the AMPK-ULK1 pathway and IRE1-JNK pathway. The IRE1-JNK signaling cascade mediates ER stress to regulate autophagy and cellular apoptosis (Hu et al., 2014). Additionally, Kaempferol can inhibit G9a expression in GC cells while upregulating LC3B-II levels to further activate autophagy (Kim et al., 2018b). Isoliquiritigenin (ISL), a natural chalcone compound primarily isolated from licorice roots, Huang et al. developed ISL-17 as the most potent ISL analog with anti-GC activity which enhances the rate of autophagy in SGC-7901 cells. The induction mechanism of autophagy is mainly associated with the PI3K/AKT/mTOR pathway (Huang et al., 2020). Zhang et al. also discovered that ISL can mediate the inhibition of proliferation, invasion, and metastasis in GC cells through autophagy (Zhang et al., 2018). Co-administration of Liquiritin (LIQ) from licorice with DDP in xenograft GC mice significantly induces apoptosis and autophagy. LIQ reduces the expression of cell cycle-related proteins in cancer cells, increases the expression of p53, LC3B, and Beclin-1, and enhances caspase-8/9/3 and PARP cleavage. Therefore, the combination therapy involving these two drugs holds significant potential for improving drug resistance while synergistically enhancing anticancer effects as well as reducing tumor volume and weight (Wei et al., 2017). Cynaroside (Cy) has a wide range of pharmacological effects such as hepatoprotective, anti-diabetic, anti-inflammatory, and anti-cancer, and Ji et al. proposed that the MET/AKT/mTOR axis may be one of the important targets for Cy to regulate various biological processes such as GC cell proliferation, apoptosis, autophagy, and invasion to exert anti-cancer properties (Ji et al., 2021). Long-term exposure to N-methyl-N-nitro-N-nitrosoguanidine (MNNG) has been identified as a crucial risk factor for GC due to its promotion of proliferation, migration through inflammation, oxygen stress, autophagy, and EMT. Studies have found that Hesperidin from citrus fruits can activate autophagy and inhibit PI3K/AKT pathway, EMT and cell proliferation, reverse the carcinogenic potency of MNNG, and provide a new approach for early intervention and treatment of GC (Liang et al., 2022). Wang et al. discovered that the anticancer mechanism of Sacroflavonoside primarily involves the regulation of apoptosis and autophagy-related proteins, including Bax, Caspase-3, Beclin-1, LC3-II, ultimately leading to apoptosis and autophagic cell death in GC cells (Wang Q. et al., 2021). Different from the above NPs, Isorhamnetin (ISO) demonstrates an inhibitory effect on autophagy in treating GC by targeting the PI3K/AKT/mTOR pathway to reverse hypoxia-induced protective autophagy in MKN-28 cells and promote mitochondria-mediated cell apoptosis. The researchers also emphasize that this is a significant but not exclusive mechanism through which ISO exerts its anti-tumor properties, suggesting untapped potential for ISO as an effective anticancer agent (Li C. et al., 2021).

Mitochondrial autophagy acts as a driving force in cancer progression and metastasis by influencing the fate of tumor cells. HIF-1α activates downstream protein Bcl-2/adenovirus E1B 19-kDa interacting protein (BNIP3) to induce mitochondrial autophagy for cell survival (Panigrahi et al., 2020). Quercetin can activate BNIP3/BNIP3L, dissociate the interaction between Beclin-1 and Bcl-2/Bcl-xL, promote the inhibitory effect of HIF-1α on mTOR complex, mediate AKT and mTOR dephosphorylation, ultimately inducing autophagy in GC cells. Further studies have found that Quercetin-induced autophagy antagonizes its pro-apoptotic effects (Wang et al., 2011). In addition, a recent study proposed a new mechanism for Quercetin to inhibit GC cell growth by promoting ATG5-mediated autophagic ferroptosis, indicating that Quercetin has both cytotoxicity against tumors and the ability to activate protective mitochondrial autophagy (Huang et al., 2024). Myricetin, a flavonoid widely present in various fruits, vegetables, and plants including green tea, has been gradually recognized for its anti-tumor properties in GC. In AGS cells, Myricetin induces apoptosis and cell-protective autophagy by modulating the PI3K/AKT/mTOR pathway. The number of apoptotic bodies and autolysosomes exhibits a dose-dependent positive correlation with Myricetin (Han et al., 2022). Similarly, other flavonoids such as Nobiletin, Pectolinarigenin, and Procyanidin B2 have also demonstrated their ability to induce cell apoptosis and autophagy through the PI3K/AKT/mTOR pathway to exert similar anti-GC effects. Notably, Pectolinarigenin-induced autophagy does not rely on Beclin-1 (Moon and Cho, 2016; Lee et al., 2018; Li Y. et al., 2021). Autophagy initiation in tumor hypoxic environments may facilitate the proliferation and rapid progression of cancer stem cells (Mukhopadhyay et al., 2022). Diosmetin, a natural flavonoid present in citrus fruits and certain medicinal herbs, acts as a modulator targeting multiple signaling pathways in cancer. Pan et al. demonstrated that Diosmetin induces apoptosis by activating the PI3K/AKT/FOXO1 signaling pathway in HGC-27 cells, while also triggering protective autophagy through activation of the MAPK/JNK pathway in cancer cells. This effect is primarily characterized by promoting phosphorylation of ERK1/2, p38, and JNK; upregulating the expression of autophagy-related protein LC3B; significantly increasing levels of pro-apoptotic proteins (such as p-Bcl2, Bak, and Bax); and dose-dependently downregulating levels of anti-apoptotic proteins (including Bcl-2, Bcl-xL, and Bid) (Pan et al., 2023). Thus, flavonoids targeting autophagy inhibition play a crucial role in GC prevention and treatment due to their dual effects on determining tumor cell survival or death.

### 4.5 Saponins

Saponins, a class of compounds widely distributed in plant stems, leaves, and roots, can be categorized into triterpenoid saponins and steroidal saponins. These compounds exhibit diverse biological activities including lipid-lowering, antimicrobial, anti-tumor, anti-thrombotic, and immune-regulating effects (Wang X. et al., 2023; Singh et al., 2017). Ginsenoside Rg5 is an uncommon active constituent derived from Panax ginseng C.A. Meyer. In xenograft mouse models of GC cells, Rg5 activates the ROS-mediated MAPK pathway to induce apoptosis and autophagy without causing organ toxicity. This effect is demonstrated by dose-dependent upregulation of ATG5, ATG12, Beclin-1 expression; downregulation of p62 protein expression; promotion of caspase-3 and PARP cleavage (Liu and Fan, 2019). Han et al. have shown that Ginsenoside Rh2 and Ginsenoside PPD exert cytotoxicity on HGC-27 cells by inhibiting autophagic flux through upregulation of LC3-II and p62 expression while increasing lysosomal pH and membrane rupture. This leads to mitochondrial damage, lysosomal dysfunction, and blockade of the autophagy pathway to inhibit cancer cell proliferation (Han et al., 2020). Known oncogene Src plays an important role in cancer invasion, metastasis, and other progression events (Šušva et al., 2000). Recent studies have found that the molecular mechanism of PPD promoting autophagic cell death in GC involves not only the activation of related genes such as ATG5, ATG7, and Beclin-1 but also a close relationship with inhibiting Src activity (Song C. et al., 2023). Astragaloside IV (AS-IV), one of the main active ingredients extracted from the traditional Chinese herb Astragalus, has been found to protect gastric mucosal tissue in MNNG-induced precancerous rat models. Its potential mechanism is closely related to reducing the Bcl-2/Bax ratio, inhibiting Bcl-xL, p53, Beclin-1, p62, ATG5 and ATG12 expression while upregulating caspase3 levels to restore a balanced relationship between cell apoptosis and autophagy (Cai et al., 2018). Saikosaponin D (SSD), an active monomer derived from traditional plant Bupleurum chinense DC., not only synergistically promotes GC cells apoptosis with DDP but also induces autophagic cell death by regulating LC3-B and p62 expression through inhibition of I kappa B kinase (IKK) β/NF-κB pathway. Furthermore, the inhibition of apoptosis does not affect SSD’s regulation on autophagy-related proteins. However, Hu did not further investigate whether SSD-induced autophagy affects tumor cell apoptosis,but it can still be proven that co-administration of SSD with DDP enhances chemotherapy sensitivity in GC cells by inducing both apoptosis and autophagy (Hu et al., 2021a). Previous studies have reported significant therapeutic effects of the natural steroidal saponin Daucosterol in cancer treatment, neuroprotection, hepatoprotection, and immune regulation (Zhang F. et al., 2023; Gao P. et al., 2019; Jiang et al., 2015). Zhao et al. first demonstrated that Daucosterol induces autophagy in GC cells both *in vitro* and *in vivo* through a ROS-dependent mechanism, exerting anti-tumor activity independent of apoptosis (Zhao et al., 2015). We have known that cancer cells employ autophagy to overcome stress conditions such as nutrient deprivation and hypoxia for survival and metastasis, which significantly contributes to poor treatment outcomes for cancer patients. Autophagy plays a pivotal role in these processes (Yang et al., 2020). Li proposed that the monomeric saponin DT-13 extracted from Liriope muscari combined with nutrient deprivation can induce substantial morphological changes towards apoptosis specifically in human gastric cancer BGC-823 cells. This effect is achieved by promoting the expression of LC3-II to trigger destructive autophagy within the cancer cells. Notably, no evident cytotoxicity was observed when DT-13 and nutrient deprivation were administered separately. Thus combining starvation therapy with autophagy regulation may represent a promising approach for enhancing the efficacy of DT-13-based anti-gastric-cancer treatment (Li et al., 2016). The normality of the p53 gene is closely related to tumor occurrence and metastasis (Hu et al., 2021b). Xu et al. discovered that A-24 derived from Allium plant species induces apoptosis in GC cells independently of p53 and inhibits metastasis. However, overexpression of p53 in GC cells significantly reduces the level of autophagy induced by A-24 through the PI3K/AKT/mTOR pathway. Nevertheless, both wild-type and defective p53 mice show induction of autophagy by A-24, indicating that while not necessary for inducing autophagy in GC cells by A-24, p53 negatively regulates its levels (Xu et al., 2021d). Fully considering the p53 phenotype of patients is one of the influencing factors for the selection of A-24 to exert anti-tumor efficacy in clinically targeted autophagy. Polyphyllin B is a natural steroidal saponin extracted from Rhizoma paridis. It has been reported to possess anticancer activity by participating in various cell death pathways. Firstly, it induces apoptosis and reduces cell viability, inhibiting GC cell migration and invasion. Additionally, Polyphyllin B treatment increases cellular lipid peroxidation levels, Fe2+ accumulation, and suppresses the activity of GPX4, a negative regulator of ferroptosis, thereby inducing iron-dependent cell death. It also promotes nuclear receptor coactivator 4-dependent ferritinophagy by regulating iron transport in GC cells. Importantly, Polyphyllin B exhibits no side effects on normal host cells while mediating its cytotoxic effects on tumor cells (Hu C. et al., 2023). Another active component, Polyphyllin I, induces autophagy by inhibiting the PDK1/AKT/mTOR signaling pathway and regulates the expression of autophagy-related markers (LC3-II and p62). Furthermore, it increases apoptotic rates in HGC-27 cells by altering nuclear morphology characteristics (He et al., 2019). Xiang et al. proposed that Polyphyllin VII exhibits potential as an effective anticancer drug by inducing autophagy-dependent ferroptosis through the inhibition of the T-lymphokine-activated killer cell-originated protein kinase (TOPK)/ULK1 signaling pathway in GC cells. Additionally, Polyphyllin VII enhances the degradation of ferritin heavy chain 1 (FTH1), a crucial regulator of iron metabolism, leading to the release of Fe2+ and lipid peroxidation-mediated ferroptosis in GC cells (Xiang et al., 2023)。Akebia saponin PA, a natural triterpenoid saponin isolated from Dipsacus asperoides, induces autophagy in various GC cell lines characterized by elevated levels of LC3-II but selectively triggers apoptosis marked by cleaved caspase-3 specifically in AGS cells. Autophagy partially or completely regulates its own induction of cellular apoptosis through subfamilies JNK, p38, and ERK within the MAPK pathway, suggesting a collaborative relationship between autophagy and apoptosis in antitumor mechanism of Akebia saponin PA primarily associated with inducing autophagic cell death in GC cells (Xu et al., 2013).

Jaspine B and its derivatives, derived from marine natural products, have gained significant attention due to their potential anticancer activity. Although it has been proposed that Jaspine B induces cytotoxicity and cytoplasmic vacuolization in GC cells, this cell death mechanism does not rely on apoptosis, autophagy or necrosis pathways (Cingolani et al., 2017). Interestingly, Xu et al. found that by altering the alkyl chain of Jaspine B to obtain its derivative C-2 and treating three different GC cells, the levels of pro-apoptotic proteins Bax and Cleaved-caspase9 were upregulated while the levels of anti-apoptotic proteins Bcl-2 and Bcl-xL were downregulated, inducing apoptosis in GC cells and exerting anti-proliferative activity. Additionally, C-2 induced autophagy by increasing the expression of proteins such as Beclin-1, LC3, and ATG12. The autophagy inhibitor LY294002 increased the death rate of GC cells. Further research revealed that C-2-mediated autophagy antagonized self-induced cell apoptosis through triggering the p62/Keap1 (Kelch-like ECH-associated protein 1)/Nrf2 cascade reaction. Moreover, the efficacy and sensitivity of C-2 in inhibiting tumors were also associated with different cell line differentiation types (Xu et al., 2022). Toxicarioside N (Tox N) derived from Antiaris toxicaria (Pers.) seeds was found to induce apoptosis in SGC-38 cells by activating MAPK signaling pathway; however, this pro-apoptotic effect could be weakened by its own inhibition on protective autophagy induced via AKT/mTOR pathway (Zhao et al., 2018a; Zhao et al., 2018b). Similarly, the steroidal saponin C21 extracted from Marsdenia induces protective autophagy in GC cells by inhibiting the PI3K/AKT/mTOR signaling pathway, while activating the PTEN/AKT/mTOR signaling pathway to induce apoptosis of tumor cells (Li K. et al., 2020). T-17 is a spiral steroidal saponin derived from the traditional Chinese herb Tupistra chinensis Baker, which demonstrates cytotoxicity against SGC-7901 and AGS cells through JNK-mediated apoptosis and activation of autophagy. Further investigations have revealed that inhibition of autophagy by 3-methyladenine (3-MA) significantly reverses T-17-induced cytotoxicity, suggesting a protective role played by autophagy (Xu J. et al., 2020). Xu et al. proposed that T-17 triggers cell apoptosis and autophagy by elevating intracellular ROS levels and downregulating HMGB1 in a p53-independent manner. However, it should be noted that p53 is crucial for the ability of T-17 to inhibit AGS cell migration. Mice lacking or with mutated p53 genes exhibit a significantly increased susceptibility to tumor metastasis, underscoring the importance of comprehending anti-gastric cancer mechanism for clinical applications (Xu et al., 2021e).

### 4.6 Polysaccharids

Natural polysaccharides have broad-spectrum pharmacological activity and low cytotoxicity. Their anti-tumor mechanisms mainly include: (a) prevention of tumor occurrence (Randeni and Xu, 2024); (b) enhanced immune activity through combined application with chemotherapy drugs (Han et al., 2021); (c) direct antagonism against tumors through regulation of tumor cell apoptosis and autophagy (Senthilkumar et al., 2013); (d) inhibition of tumor metastasis (Varghese et al., 2019). Currently, research on the anti-tumor mechanisms of polysaccharides primarily focuses on inducing cell apoptosis, regulating autophagy, and related signaling pathways. Increasingly, natural polysaccharides have been proven to possess anti-gastric cancer activity *in vitro* experiments, animal studies, and clinical trials (Li J. et al., 2023). The polysaccharides extracted from the newly developed sporoderm-removed spores of G. lucidum(RSGLP) upregulate the expression of autophagy-related proteins, LC3-II and p62, while inhibiting autophagosome-lysosome fusion in AGS cells. This leads to the accumulation of autophagosomes and disruption of autophagic flux, inducing apoptosis in AGS cells. Unlike most NPs that induce autophagy, RSGLP functions as an autophagy inhibitor but still exhibits anti-tumor effects by inhibiting tumor cell viability and promoting apoptosis in GC patients’ treatment (Zhong et al., 2021). Astragalus polysaccharide (APS), a monomeric component extracted from Radix astragali mongolici, has demonstrated significant anti-tumor bioactivity (Li W. et al., 2020). Compared with apatinib alone (a third-line drug for the treatment of GC), the combination of APS and apatinib induces the conversion of LC3-I to LC3-II and induces an increase in autophagy levels by promoting the conversion of LC3-I to LC3-II of autophagosome biosynthesis, and blocking autophagy is beneficial to reverse the enhancement of autophagy induction on GC cell viability and the inhibition of apoptosis. In addition, APS inhibited the overactivation of p-AKT and the overexpression of Matrix metalloproteinase-9 (MMP-9), attenuating the promotion of tumor cell proliferation and migration, indicating that APS-induced autophagy and apoptosis assist each other in the anti-GC effect (Wu et al., 2018). Derived from marine brown algae, Fucoidan exhibits promising prospects for applications in kidney diseases, cancer, and inflammatory reactions (Shi et al., 2024; Zahan et al., 2022; Yu et al., 2024). Zhu et al. demonstrated through cellular experiments that Fucoidan induces autophagy and apoptosis in AGS cells synergistically, leading to cytotoxic effects characterized by increased accumulation of Beclin-1, conversion from LC3-I to LC3-II, and downregulation of Bcl-2 and Bcl-xL protein expression (Park et al., 2011). Fucoxanthin (a natural carotenoid) belonging to the same genus as Fucoidan has also been shown to possess similar anti-tumor effects and mechanisms of action (Zhu et al., 2018). In summary, based on existing research findings, natural polysaccharides may modulate autophagy in gastric cancer by either inducing or inhibiting autophagic flux while demonstrating significant therapeutic potential in preventing cancer initiation or impeding tumor growth and progression. NPs targeting autophagy improve GC is illustrated in Table 2.

**TABLE 2:** NPs targeting autophagy improve GC. The subsequent table categorizes NPs that exert prosurvival or prodeath effects on cancer cells and lists the functional states and molecular mechanisms through which autophagy is regulated by terpenoids, polyphenols, alkaloids, flavonoids, saponins, and polysaccharides. As a potential anti-cancer therapeutic agent or as an adjunct to new chemotherapy drugs, these NPs can modulate autophagy levels thereby enhancing efficacy or reducing side effects. ↑ indicates activate or increase; ↓ indicates inhibite or reduce. RSGLP, polysaccharides extracted from the newly developed sporoderm-removed spores of G. lucidum.

Classification	Function on tumors	Source	Compounds	Cell lines	Mechanism	Reference
Terpenoids	Prodeath	Licorice	18β-Glycyrrhetinic acid	GES-1, AGS, HGC-27	↑miR-345-5p/TGM2; ↑TGM2, p62; ↓LC3-II,ULK1,AMPK	Li et al. (2023b)
				AGS	↑miR-328-3p/STAT3	Yang et al. (2023)
				AGS	↓MAPK; ↓KRAS, ERK1, ERK2	Li et al. (2023c)
			Glycyrrhizin	AGS	a.↑LC3B-Ⅱ; ↓LAMP1, p62, HMGB1, ROS b.↓H.pylori	Khan et al. (2023)
		Taxus sp	Paclitaxel	BGC823	↓p62	Yu et al. (2017)
			Albumin paclitaxel	AGS	↑AMPK, p-ULK1, LC3-II/LC3-I, Bcl-2, Bax; ↓SQSTM1/p62, Beclin-1	Cheng et al. (2022)
			Paclitaxel + OBP-401	GCIY, KATOIII	↑PARP, adenoviral E1A; ↓p62	Ishikawa et al. (2020)
		Ganoderma lucidum	Methanolic extract of Ganoderma lucidum fruiting body	AGS	↑LC3-II; ↓p62	Oliveira et al. (2014)
		Tripterygium wilfordii Hook F	Celastrol	AGS, YCC-2	↑AMPK; ↓AKT/mTOR. ↑LC3-I, LC3-II, ATG5, ATG7, Beclin-1, cytochrome C, Bax; ↓Bcl-2,Bcl-xl, caspase-3/9/8	Lee et al. (2014)
		Rabdosia rubescens	Jiyuan Oridonin A	HGC27, MGC803, AGS	↑LC3B, cleaved caspase-3, cleaved PARP	Wang et al. (2021a)
			a2	HGC-27, MGC-803, BGC-823, AGS, GES1	a. ↑PARP, caspase-9/3; ↓Bcl-2 b. ferroptosis, ↓GPX4; ↑SLC7A11, ROS, ferrous iron accumulation c. superior pharmacokinetic characteristics	Liu et al. (2021)
			Jaridon 6	MGC803, PTX	↓PI3K/AKT ↓SIRT1, p62, β-actin; ↑LC3B-Ⅱ	Fu et al. (2021a)
		Cucurbitaceae	Cucurbitacin B	SGC7901/DDP	↓CIP2A/PP2A/mTORC1 ↓P-gp, HIF-1α; ↑caspase-9/3, cleaved PARP, LC3-II, Beclin-1	Liu et al. (2017)
			Cucurbitacin D/E/I	AGS	↑LC3	JafargholizadeH et al. (2018)
		Perilla frutescens	Perillaldehyde	MFCs(mouse), GC9811-P	↑AMPK. ↑p-ULK1, Beclin-1, LC3-II, cathepsin, caspase-3, p53	Zhang et al. (2019)
		Rabdosia japonica	Glaucocalyxin B	SGC-7901	↓NF-κB, MAPK/ERK; ↑Bax/Bcl-2, cleaved caspase-3, cleaved PARP, LC3A/B-II, ROS	Ur Rahman et al. (2017)
		Curcuma zedoaria	Germacrone	SGC7901, MGC803	↑cleaved caspase-3, Bax/Bcl-2, LC3-II/LC3-I, p53, p62; ↓JAK2/STAT3, Bcl-2	Fang et al. (2020)
		Artemisia annua	Dihydroartemisinin	SGC7901/DDP	↓PI3K/AKT/mTOR. ↑caspase-8/9/3, Beclin-1, LC3-II; ↓P-gp	Zhang et al. (2022)
	Prosurvival	Nigella sativa	Thymoquinone	HGC-27, MKN-45, GES-1	↓PI3K/AKT/mTOR. ↑LC3B-II, ATG5, cleaved PARP, cleaved caspase-9/3, Bax; ↓Bcl-2, p62	He et al. (2023)
		Rhizoma zedoariae	β-Elemene	MGC803, SGC7901	↓PI3K/AKT/mTOR/p70S6K1 ↑LC3-II, ATG5-ATG12 conjugated protein, cleaved PARP; ↓survivin	Liu et al. (2011)
		Rhodiola rosea	Salidroside	AGS	↓PI3K/AKT/mTOR. ↓Bcl-2, Bcl-xL; ↑Bax,Beclin-11,LC3-II	Rong et al. (2020)
		Tripterygium wilfordii	Triptolide	AGS, IM95	↓PRDX2, p62; ↑LC3-II, ROS, ER stress	Chen et al. (2024a)
		Anemone raddeana regel	Raddeanin A	SGC-7901	↑p38 MAPK; ↓mTOR. ↑LC3II, Beclin-1, ATG3, ATG5, ATG7, Bax, cleaved PARP, cleaved caspase-3; ↓Bcl-2,Bcl-xL	Teng et al. (2016)
Polyphenols	Prodeath	Curcuma longa	Curcumin	SGC-7901, BGC-823	↓PI3K/AKT/mTOR. ↑Beclin-1,ATG3,ATG5,LC3-II/LC3-I,p53,Bax,cleaved PARP, cleaved caspase-3, Fe^2+^,MDA,ROS,ACSL4; ↓Bcl-2,Bcl-xL,GSH,SLC7A11,GPX4	Fu et al. (2018), Xiao et al. (2022), Zheng et al. (2024b)
		Mallotus philippinensis	Rottlerin	SGC-7901, MGC-803	↑LC3-II; ↓mTOR, Skp2	Song et al. (2018)
		Resveratrol	Piceatannol	SGC790, BGC823, MKN28, MGC803, HGC27, and AGS	↑LC3-II/LC3-I, caspase-9/3; ↓SQSTM1/p62, p-Beclin-1, Bcl-2	Huangfu et al. (2023)
		Echinacea purpurea	Chicoric acid	SGC-7901, MGC-803	↑AMPK ↑ER stress, LC3-II, Beclin-1, ATG5, cleaved PARP, cleaved caspase-3; ↓p62	Sun et al. (2019)
		Grapes, red wine, peanuts, blueberries and so on	Resveratrol	HGC-27	↑LC3-II, ceramide	Signorelli et al. (2009)
	Prosurvival	Ellagitannin	Urolithin A	HGC-27, MKN-45	↓PI3K/AKT/mTOR. ↑Bax, LC3-II/LC3-I, cleaved caspase-3; ↓Bcl-2, p62	Zhang et al. (2023a)
		Phyllanthus niruri L	Corilagin	SGC7901, BGC823, GES-1, HT-29, HeLa	↓procaspase-8/9/3; ↑LC3-II,cleaved PARP,ROS	Xu et al. (2019)
Alkaloids	Prodeath	Cruciferous vegetables	3,3′-Diindolylmethane	BGC-823, SGC-7901	a.↑ATG5, LC3-II; ↓miR-30e b.↑Ca^2+^/AMPK/ER stress; ↑STIM1, CHOP, Bax, cleaved caspase-3, LC3-II/LC3-I; ↓Bcl-2	Ye et al. (2016), Ye et al. (2021)
		Stephania tetrandra S	Tetrandrine	HGC-27	↓PI3K/AKT. ↑Beclin-1, LC3-II, p62, cleaved PARP, cleaved caspase-9/3	Bai et al. (2018)
		Sophora	Matrine	SGC-7901	↑Beclin-1, Bax	Zhang et al. (2011)
		Evodia rutaecarpa	Evodiamine	SGC-7901	↑Beclin-1, Bax; ↓Bcl-2	Rasul et al. (2012)
		Rhizoma coptis	Berberine	-	a. ↓MAPK, P13K/AKT/mTOR; ↓Bcl-2, cytochrome C, mitochondrial membrane potential, p-BAD; ↑caspase-3, cleaved PARP b. ↓AMPK, NF-κB, *H. pylori*	Liu et al. (2022)
		Coptidis rhizoma	Magnoflorine	MGC803, BGC823, SGC7901, GES-1	↓P13K/AKT/mTOR; ↑JNK signaling pathway ↑ROS, LAMP1, p62, LC3B-II, caspase-3, cleaved PARP, Bax; ↓Bcl-2	Sun et al. (2020)
		Amaryllidaceae	Narciclasine	BGC-823, MGC-803, GES-1, MKN28,SGC-7901	↓AKT/mTOR. ↑LC3-II, ATG-5, Beclin-1, Bax, cleaved-PARP, cleaved-caspase-3/8/9, cytochrome C ↓Bcl-2,p62	Yuan et al. (2021)
		Sophorae flavescentis	Sophocarpine	MKN45, BGC-823	↓PTEN/PI3K/AKT. ↓p62, Bcl-2; ↑LC3-I,LC3-II, caspase-3, Bax	Huang et al. (2019)
		Solanum nigrum Linn	Solanine	SGC-7901, MGC-803, GES-1	↓AAMDC/MYC/ATF4/Sesn2 ↑caspase-3, Bax; ↓LC3-II/LC3-I, Bcl-2	Tang et al. (2023)
	Prosurvival	Catharanthus roseus	Vincristine	SGC-7901, BGC-823	↑HMGB1, MCL-1, LC3-II, Beclin-1	Zhan et al. (2012)
		Chaetomium	Chaetocin	HGC-27, NCI-N87,293T, HeLa	↑LC3B-II, cleaved-PARP, cleaved caspase-3/8/9, Bax; ↓p62,mitochondrial membrane potential, Bcl-2	Liao et al. (2019)
Flavonoids	Prodeath	Citrus	Naringin	AGS	↓PI3K/AKT/mTOR. ↑Beclin-1, LC3B, ERK1/2, JNK, p38 MAPK, LAMP1, BH3, ROS; ↓Bcl-xL	Raha et al. (2015), Raha et al. (2020)
				SNU-1, GES-1	↓PI3K/AKT. ↑Beclin-1, LC3-II/LC3-I, caspase-3, Bax; ↓Bcl-2, p62	Xu et al. (2021b)
		Most vegetables and fruits	Apigenrin	AGS, SNU-638	↑mTOR/AMPK/ULK1; ↓PI3K/AKT/mTOR. ↑ATG-5, Beclin-1, LC3-II, LDH, caspase-3/9, ER stress; ↓p62,HIF-1,Ezh2	Kim and Lee (2021), Kim et al. (2020a)
		Scutellaria baicalensis	Baicalein	MGC-803, HGC-27, SGC-7901, SGC-7901/DDP	↓AKT/mTOR, Nrf2/Keap 1; ↑LC3,Beclin-1; ↓p62	Li et al. (2020b)
			Wogonin	BGC-823	↑LC3-II, p-JNK, peroxynitrite; ↓p-ULK1,mitochondrial membrane potential	Hong et al. (2018)
		Silybum marianum L	Silymarin-SeNPs	AGS	↓PI3K/AKT/mTOR ↑Beclin-1, LC3-II, Bax/Bcl-2, cytochrome C, caspase-3/9; ↓p62	Mi et al. (2022)
		Cinnamomum cassia	Cinnamaldehyde	SNU-638, SNU-216, AGS, NCI-N87, MKN-45, MKN-74	↑PERK/ATF4/CHOP, mTOR/AMPKα/ULK1 ↑Beclin-1, ATG5, LC3B, cleaved caspase-3/9, Ca^2+^, ER stress; ↓p62, Bcl-2, G9a	Kim (2022)
		Kaempferol galanga L	Kaempferol	AGS, SNU-216, NCI-N87, SNU-638, NUGC-3, MKN-74	↑IRE1/JNK/CHOP, AMPK-ULK1 ↑LC3-II/LC3-I, Beclin-1, ATG5, ER stress, cleaved caspase-3/9; ↓p62, G9a, Bcl-2	Kim et al. (2018b)
		Licorice	Isoliquiritigenin-17	SGC-7901, BGC-823, GES-1	↓PI3K/AKT/mTOR. ↑LC3B-II, Beclin-1, cleaved-PARP, ax, OS; ↓Bcl-2, 62	Huang et al. (2020)
			Isoliquiritigenin	MKN28, GES-1	↓PI3K/AKT/mTOR. ↑LC3-II/LC3-I, Beclin-1, ax, aspase-3; ↓p62, cl-2	Zhang et al. (2018)
			Liquiritin	SGC7901/DDP	↑LC3B-II/LC3-I, Beclin-1, cleaved caspase-3/8/9; ↓p62	Wei et al. (2017)
		Honeysuckle	Cynaroside	HGC27, MKN45, SGC7901	↓MET/AKT/mTOR. ↑cleaved-PARP, cleaved caspase-3	Ji et al. (2021)
		Citrus fruits	Hesperidin	-	↑PI3K/AKT. ↑Beclin-1, LC3-II/LC3-I, ATG5, E-cadherin; ↓vimentin, PCNA	Liang et al. (2022)
		Artemisia sacrorum	Sacroflavonoside	MKN-45	↑Bax, caspase-3, Beclin-1, LC3-II; ↓Bcl-2	Wang et al. (2021b)
		Sea buckthorn	Isorhamnetin	MKN-28, GES-1	↓PI3K/AKT/mTOR. ↑Bax/Bcl-2, caspase-3, LC3-II/LC3-I, Beclin-1; ↓p62	Li et al. (2021a)
	Prosurvival	Most vegetables and fruits	Quercetin	AGS, MKN28	↑BNIP3/BNIP3L; ↓AKT/mTOR ↑LC3-II/LC3-I, Beclin-1, ATG7, ATG12-ATG5 conjugated protein, HIF-1α	Wang et al. (2011)
				AGS, MKN45, MKN7, TMK1, GES-1	↓TFR1, GSH; ↑membrane density, ROS, lipid peroxidation,Fe^2+^ accumulation,ATG5,LC3B,Beclin-1	Huang et al. (2024)
		Bayberry and so on	Myricetin	AGS	↓PI3K/AKT/mTOR. ↑LC3-II/LC3-I, Beclin-1, cleaved-PARP, Bax; ↓Bcl-2	Han et al. (2022)
		Citrus fruits	Nobiletin	SNU-16	↓AKT/mTOR. ↑LC3B-II/LC3B-I, caspase-4, cleaved-PARP, ER stress; ↓p62	Moon and CHO (2016)
		Cirsium chanroenicum	Pectolinarigenin	AGS, MKN28	↓PI3K/AKT/mTOR. ↑LC3-II/LC3-I, caspase-3/7, cleaved-PARP; ↓Beclin-1	Lee et al. (2018)
		Most vegetables and fruits	Procyanidin B2	BGC-823, SGC-7901	↓AKT/mTOR. ↑caspase-3/9, Beclin-1, ATG5, LC3-II/LC3-I	Li et al. (2021b)
		Citrus fruits and so on	Diosmetin	HGC-27	↓PI3K/AKT/FOXO1; ↑MAPK/JNK. ↓Bcl-2, Bcl-xL, Bid; ↑cleaved-PARP, cleaved caspase-3,Bax,Bak,p-Bcl2	Pan et al. (2023)
Saponins	Prodeath	Panax ginseng C.A. Meyer	Ginsenoside Rg5	SGC-7901, BGC-823	↑MAPK. ↑ATG5, ATG12, Beclin-1, LC3B-II, cleaved pro-caspase-9, cytochrome C, Bax, cleaved-PARP, ROS, p-JNK, p-p38, p-ERK; ↓Bcl-2, p62	Liu and Fan (2019)
			Ginsenoside Rh2	HGC-27	↑LC3-II, p62, ROS	Han et al. (2020)
			Ginsenoside PPD			
		Astragalus	Astragaloside IV	-	↑caspase-3; ↓Bcl-2/Bax,Bcl-xL,p53,Beclin-1, ATG5,ATG12, p62,LC3, Ambra1	Cai et al. (2018)
		Radix bupleuri	Saikosaponin D	SGC-7901, SGC-7901/DDP	↓IKK β/NF-κB ↑cleaved caspase-3, LC3B; ↓p62	Hu et al. (2021a)
		Paris polyphylla J.E. Smith	Daucosterol	MCF-7, AGS, BGC823, MGC803	↑ROS, LC3-II, Beclin-1	Zhao et al. (2015)
		Liriope muscari	DT-13	BGC-823, A549	↓PI3K/AKT/mTOR. ↑LC3-II, Beclin-1, ATG7, ATG3; ↓caspase-3/9	Li et al. (2016)
		Allium chinense	A-24	AGS, KATO-III	↓PI3K/AKT/mTOR. ↑LC3-II, cleaved PARP-1, cleaved caspase-3, Bax, ROS; ↓Bcl-2, p53	Xu et al. (2021d)
		Rhizoma paridis	Polyphyllin B	NUGC-3, MKN-1, MKN-45, HGC-27, NUGC-4	↓GPX4; ↑LC3B, TFR1, NOCA4, FTH1, ferroptosis	Hu et al. (2023b)
			Polyphyllin I	HGC-27	↓PDK1/AKT/mTOR. ↑LC3-II/LC3-I; ↓p62	He et al. (2019)
			Polyphyllin VII	AGS, NCI-N87, GES-1, BGC823, MGC803, MKN74, HGC27	↑ULK1, ferroptosis, LC3-II; ↓TOPK, p62, FTH1	Xiang et al. (2023)
		Dipsacus asperoides	Akebia saponin PA	AGS	↓PI3K/AKT/mTOR, MAPK; ↑AMPK/mTOR. ↑LC3-II, caspase-3, cleaved PARP-1	Xu et al. (2013)
	Prosurvival	Marine sponges	C-2	293T, MGC803, HGC27, SGC7901, GES-1	↑JNK/ERK, p62/Keap1/Nrf2 ↑Beclin-1, LC3, ATG12, ATG3, cleaved-caspase3, cleaved-PARP, p62	Xu et al. (2022)
		Antiaris toxicaria	Toxicarioside N	SGC-7901	↓AKT/mTOR. ↑LC3-II, Beclin-1, cleaved caspase-3, cleaved-PARP; ↓SQSTM	Zhao et al. (2018a)
				SGC-7901	↑p38MAPK signaling pathway ↑cleaved caspase-3/9, cleaved-PARP, Bax/Bcl-2	Zhao et al. (2018b)
		Marsdenia	C21	BGC-823, SGC-7901, AGS	↓PI3K/AKT/mTOR. ↑LC3-II, Beclin-1, ATG-5, cleaved-PARP, Bax, ROS; ↓Bcl-2, p-AKT	Li et al. (2020c)
		Tupistra chinensis Baker	T-17	SGC-7901, AGS	↑JNK signaling pathway ↑Beclin-1, Bax, cleaved PARP-1, cleaved caspase-3; ↓SQSTM1/p62, Bcl-2	Xu et al. (2020b)
Polysaccharides	Prodeath	Ganoderma. lucidum	RSGLP	AGS, MKN28, NCI-N87, GES-1	↓Bcl-2, pro-caspase-3; ↑cleaved-PARP, LC3-II, p62	Zhong et al. (2021)
		Radix Astragali Mongolici	Astragalus polysaccharide	HUVEC, AGS	↓AKT signaling pathway ↓MMP-9; ↑LC3-II/LC3-I	Wu et al. (2018)
		Brown algae	Fucoidan	AGS	↓Bcl-2, Bcl-xL, Bid; ↑caspase-3/8/9, Beclin-1, LC3-II/LC3-I	Park et al. (2011)
			Fucoxanthin	SGC-7901	↑Beclin-1, LC3, cleaved caspase-3; ↓Bcl-2	Zhu et al. (2018)

## 5 Valuation of the toxic effects of natural products

The potential of NPs to be repositioned as anticancer drugs has been controversial because, despite their broad anticancer activity, they do not produce nonspecific off-target effects on normal tissues. Ginkgolic acid has been shown to have cytotoxic, embryotoxic, neurotoxic, and enzyme system inhibitory properties, and the International Pharmacopoeia stipulates that its standard concentration must be less than 5.00 mg/kg (Dong et al., 2021). A 21-day long-term dose toxicity study confirmed that the maximum tolerated dose of 20 mg/kg for compound 45, a derivative of Wuchererine, became the turning point in the dose-efficacy-toxicity curve, and that it exhibits strong toxicity when dosed at 40 mg/kg in mice, but that it has a significant potential to overcome adriamycin resistance in hepatocellular carcinoma (Wei et al., 2024). In a study on the effect of rosemarinic acid combined with ginsenoside Rg1 on colon cancer metastasis, the combination of the two exerted a superior anti-metastatic effect than rosemarinic acid alone and did not result in increased visceral toxicity in the liver, stomach, or colon (Liu et al., 2024). Clinical trials have found that ginsenoside Rg3 promotes cytotoxicity and apoptosis of paclitaxel by inhibiting NF-κB signaling and regulating Bax/Bcl-2 expression, and exhibits good chemosensitization in the treatment of triple-negative breast cancer (Yuan et al., 2017). Genistein has been reported to have some reproductive toxicity, manifesting as reduced sperm counts and lower serum testosterone counts in men, and unchanged reproductive hormones in women, but with adverse effects on the course of pregnancy and more pronounced effects on male fertility (Rashid et al., 2022). Previous studies have pointed to the possibility of moderate toxic effects such as hypertension and hypokalemia with long-term licorice use, but this evidence is generally derived from case reports and is not sufficiently convincing (R et al., 2017). A toxicity trial evaluating the safety of Nonpolar extract from the root of Glycyrrhiza uralensis (NERG) demonstrated that the median lethal dose (LD50) of oral NERG exceeded 2,000 mg/kg, i.e., ingestion of up to 2,000 mg/kg of NERG by the oral route resulted in little to no acute toxicity. No organ weight differences, subchronic toxicity phenomena, or deaths were also observed in mice receiving oral doses of 50 mg/kg, 100 mg/kg, 500 mg/kg, or 1,000 mg/kg for 120 days (Kim H. et al., 2020). In summary, balancing the toxic effects and pharmacological activities of NPs is the key to optimal efficacy, which is influenced by important factors such as individual differences, tumor type and appropriate dose. In addition, the complexity of the tumor microenvironment *in vivo* and the influence of drug-drug interactions on efficacy and safety cannot be ignored. Finally, there is a need to further improve the science of clinical trial design and data analysis, which is crucial to support NPs as anticancer agents.

## 6 Conclusion and future perspective

GC holds a significant position in the spectrum of human gastrointestinal diseases, characterized by its pronounced aggressiveness and heterogeneity, which present formidable challenges related to metastasis, recurrence, and drug resistance. Autophagy plays a dual role in this context; it inhibits tumor initiation while simultaneously facilitating tumor progression, thereby exhibiting a complex, environment-dependent influence on GC. In recent years, substantial evidence has emerged regarding the safety and efficacy of NP that target autophagy regulation, elucidating their mechanisms of action and highlighting their potential as promising anticancer therapeutics. Nonetheless, there is a gap in summarising previous basic research and the mechanism of action of NPs. This review underscores the anticancer properties of NP, which primarily modulate intact autophagic flux through various pathways, including PI3K/Akt/mTOR, AMPK, p53 and MAPK. These pathways subsequently inhibit cell proliferation, regulate energy metabolism, monitor bacterial and tumor immune evasion, promote apoptosis, and induce ferroptosis. Furthermore, this paper examines the role of NPs in suppressing cancer cell invasion and metastasis, as well as their capacity to modulate chemosensitivity.

NPs present notable anti-GC advantages due to their structural diversity, low toxicity and side effects, and ready availability. Additionally, they address the limitations of single-target therapies prevalent in Western medicine by offering efficacy comparable to current combination therapies. A substantial proportion of new chemical entities developed globally are derived, directly or indirectly, from natural sources, highlighting the significant medicinal value and considerable market potential of these compounds. However, despite their promise, the clinical application of NPs as autophagy modulators in the treatment of GC continues to encounter numerous challenges. For instance, there exists a notable scarcity of resources for numerous efficacious NPs, such as paclitaxel, alongside a significant disparity between the drug yield and the administered dose necessary to exert antitumor effects (Martelli et al., 2022). Nevertheless, the rapid advancements in technologies like metabolic engineering and synthetic biology have substantially mitigated the production challenges associated with NPs, including scarcity, inefficiency, and low yield (Helf et al., 2024). These advancements have paved the way for the economic and sustainable development of pharmaceuticals. Furthermore, a primary obstacle in the development of various chemopreventive agents, such as genistein and curcumin, has been their solubility and low oral bioavailability. It is anticipated that these challenges are gradually being surmounted through solutions such as the development of nanoparticle-based delivery systems, chemical modifications, and the use of bioenhancers, which are expected to positively impact drug dosage reduction, side effect mitigation, and patient compliance (Sanidad et al., 2019; Yang et al., 2012). Additionally, the strong association between H. pylori and autophagy offers new perspectives for the treatment of GC, and previous studies have demonstrated that NPs can serve as a novel alternative strategy to counteract the development of H. pylori-I into GC (Sarkar et al., 2016). At present, there is a paucity of specific NPs that specifically target the interaction between H. pylori and autophagy in GC. Consequently, there is significant value in accelerating the discovery of additional natural products that address both *H. pylori* and autophagy, as this could expand the therapeutic landscape for GC.

While a “one drug, one target” strategy may effectively alleviate clinical symptoms, the multi-target and multi-pathway regulatory mechanisms of autophagy position it as a promising approach for personalized and comprehensive treatment of various clinical conditions. But in fact, the complexity of autophagy cannot be understood as a single regulation of the ATG genes, Beclin-1, etc., and may involve crosstalk with other cell death modes, which complicates the threshold for distinguishing between “protective” effects and its “lethal” consequences of autophagy (Zhang G. et al., 2023). Consequently, further foundational research is imperative to elucidate the bidirectional regulation of autophagy.

The animal experimental studies that examine the modulation of autophagy by TCM in the prevention and management of GC primarily rely on disease models, which do not adequately align with the holistic diagnostic and therapeutic principles of TCM. To advance the scientific understanding of the alterations in biological indicators of GC progression under the influence of NPs, it is imperative to adopt a comprehensive and multi-dimensional research methodology in future animal studies. Crucially, given that current research on the modulation of autophagy by NPs in the context of GC predominantly focuses on basic experimental studies with a paucity of clinical observational research, future efforts should prioritize the translation of preclinical findings into large-scale, multicenter, double-blind, randomized controlled clinical trials. Such trials should adhere to rigorous standardization, thorough scientific testing, and precise data collection. This approach will facilitate a comprehensive understanding of the pharmacological activities of NPs, enabling the identification and validation of NPs that function as autophagy inducers or inhibitors. This endeavor is of significant importance for bridging existing evidence gaps and expanding the conceptual framework for targeted GC treatment strategies.
